# *Streptococcus pyogenes* Sortase Mutants Are Highly Susceptible to Killing by Host Factors Due to Aberrant Envelope Physiology

**DOI:** 10.1371/journal.pone.0140784

**Published:** 2015-10-20

**Authors:** Assaf Raz, Ana-Maria Tanasescu, Anna M. Zhao, Anna Serrano, Tricia Alston, Asaf Sol, Gilad Bachrach, Vincent A. Fischetti

**Affiliations:** 1 Bacterial Pathogenesis and Immunology, The Rockefeller University, 1230 York Avenue Box 172, New York, New York, 10065, United States of America; 2 Institute of Dental Sciences, Hebrew University - Hadassah School of Dental Medicine, Jerusalem 91120, Israel; East Carolina University School of Medicine, UNITED STATES

## Abstract

Cell wall anchored virulence factors are critical for infection and colonization of the host by Gram-positive bacteria. Such proteins have an N-terminal leader sequence and a C-terminal sorting signal, composed of an LPXTG motif, a hydrophobic stretch, and a few positively charged amino acids. The sorting signal halts translocation across the membrane, allowing sortase to cleave the LPXTG motif, leading to surface anchoring. Deletion of sortase prevents the anchoring of virulence factors to the wall; the effects on bacterial physiology however, have not been thoroughly characterized. Here we show that deletion of *Streptococcus pyogenes* sortase A leads to accumulation of sorting intermediates, particularly at the septum, altering cellular morphology and physiology, and compromising membrane integrity. Such cells are highly sensitive to cathelicidin, and are rapidly killed in blood and plasma. These phenomena are not a loss-of-function effect caused by the absence of anchored surface proteins, but specifically result from the accumulation of sorting intermediates. Reduction in the level of sorting intermediates leads to a return of the sortase mutant to normal morphology, while expression of M protein with an altered LPXTG motif in wild type cells leads to toxicity in the host environment, similar to that observed in the sortase mutant. These unanticipated effects suggest that inhibition of sortase by small-molecule inhibitors could similarly lead to the rapid elimination of pathogens from an infected host, making such inhibitors much better anti-bacterial agents than previously believed.

## Introduction

The cell wall of Gram-positive pathogens is coated with numerous covalently anchored virulence factors that are critical for the establishment of infection [1–4]. These factors have an N-terminal leader sequence, directing them for translocation through the secretion channel, and a conserved C-terminal sorting signal, comprised of an LPXTG motif followed by a hydrophobic region and a few positively charged amino acids at the C-terminus [5, 6]. Translocation is halted when the C-terminal sorting signal reaches the secretion channel. At this state the LPXTG motif is exposed on the extracellular side of the membrane, the hydrophobic stretch spans the membrane, and the positively charged residues are within the cytoplasm [6, 7]. The membranal enzyme sortase then cleaves the LPXTG motif between the threonine and glycine residues, and connects the freed threonine to lipid II [2, 8, 9]. The lipid II—protein complex is then processed by penicillin binding proteins, finally attaching the protein to the wall. The C-terminal portion of the cleaved sorting signal, containing the hydrophobic region and positively charged residues, is released back into the cytoplasm [10].

*Streptococcus pyogenes* is an important human pathogen, causing over 500,000 deaths yearly [11, 12]. The most common disease conditions caused by this organism are pharyngitis and pyoderma, and these are typically self-limiting. Nevertheless, infection may become invasive, and lead to severe conditions such as toxic shock syndrome, septicemia, and necrotizing fasciitis. Additionally, streptococcal infection may lead to sequelae, including rheumatic heart disease and glomerulonephritis [13, 14].

Surface proteins are critical for colonization and infection of the host by *S*. *pyogenes*. Similar to other Gram-positive bacteria, the majority of *S*. *pyogenes* surface proteins are anchored by the housekeeping sortase, SrtA [15]. Two *S*. *pyogenes* surface proteins in particular, M protein and SfbI, have been used as models for the study of protein anchoring [16, 17]. M protein is essential in preventing opsonization and the subsequent elimination of *S*. *pyogenes* through phagocytosis, and it also interacts with a range of host factors [18–20]. SfbI is the major fibronectin binding protein of certain streptococcal strains, and is important in mediating adhesion and invasion of host cells [21–23]. M protein is anchored at the septum [24] while SfbI displays a polar localization [25]. These distinct localization patterns are governed by differences in the signal sequence of these proteins, which lead to translocation at different cellular regions [16]. A direct correlation exists between the presence of a YSIRK-G/S motif in the signal sequence of surface proteins in Gram-positive bacteria and translocation at the septum, however the YSIRK sequence itself does not appear to be the septum targeting motif [16, 26]. M protein (containing a YSIRK G/S motif) is rapidly anchored at a very narrow septal band. Conversely, SfbI (with no YSIRK G/S motif) is anchored in a gradual manner at large peripheral regions of the cell, where its accumulation over time results in polar distribution [17].

While deletion of sortase is not typically lethal, it leads to a marked reduction in the ability of various Gram-positive bacterial pathogens to colonize and infect the host [27–38]. When sortase is deleted, covalent anchoring of proteins to the cell wall is prevented, and an increased proportion of surface proteins remain as membrane-bound intermediates. Some of the trapped proteins are subsequently released into the surrounding medium or remain associated non-covalently with the cell wall [27]. In this study we aimed for a better understanding of the fate of surface proteins following deletion of *S*. *pyogenes* sortase. We found that deletion of sortase leads to accumulation of surface proteins primarily at the septum and equatorial rings, and these caused a substantial alteration of cellular morphology and physiology. These changes directly affected the fitness of the sortase mutants in the host environment, over and above the mere loss-of-function effects that are caused by the absence of anchored virulence factors.

## Results

### Deletion of *S*. *pyogenes* sortase results in morphological aberrations

During our work with the *S*. *pyogenes* sortase mutant, we noticed substantial morphological aberrations in this strain. The aim of the current study was to characterize the cause and the implications of this phenotype. An observation that proved helpful in approaching this problem was made early on. We noticed that passage of the sortase mutant in laboratory medium for several generations resulted in a return to wild type morphology. We reasoned that understanding the changes that led to a return to wild type morphology could help understand the underlying cause for the morphological aberrations. To determine whether a return to normal morphology occurs reproducibly, we passaged 10 colonies (streaked from the original stock) six times. In each passage, a small amount of the previous day’s culture was inoculated into TH+Y medium, and grown overnight. Following six passages, all the cultures displayed normal chain organization when examined by phase-contrast microscopy.

**Fig 1:**
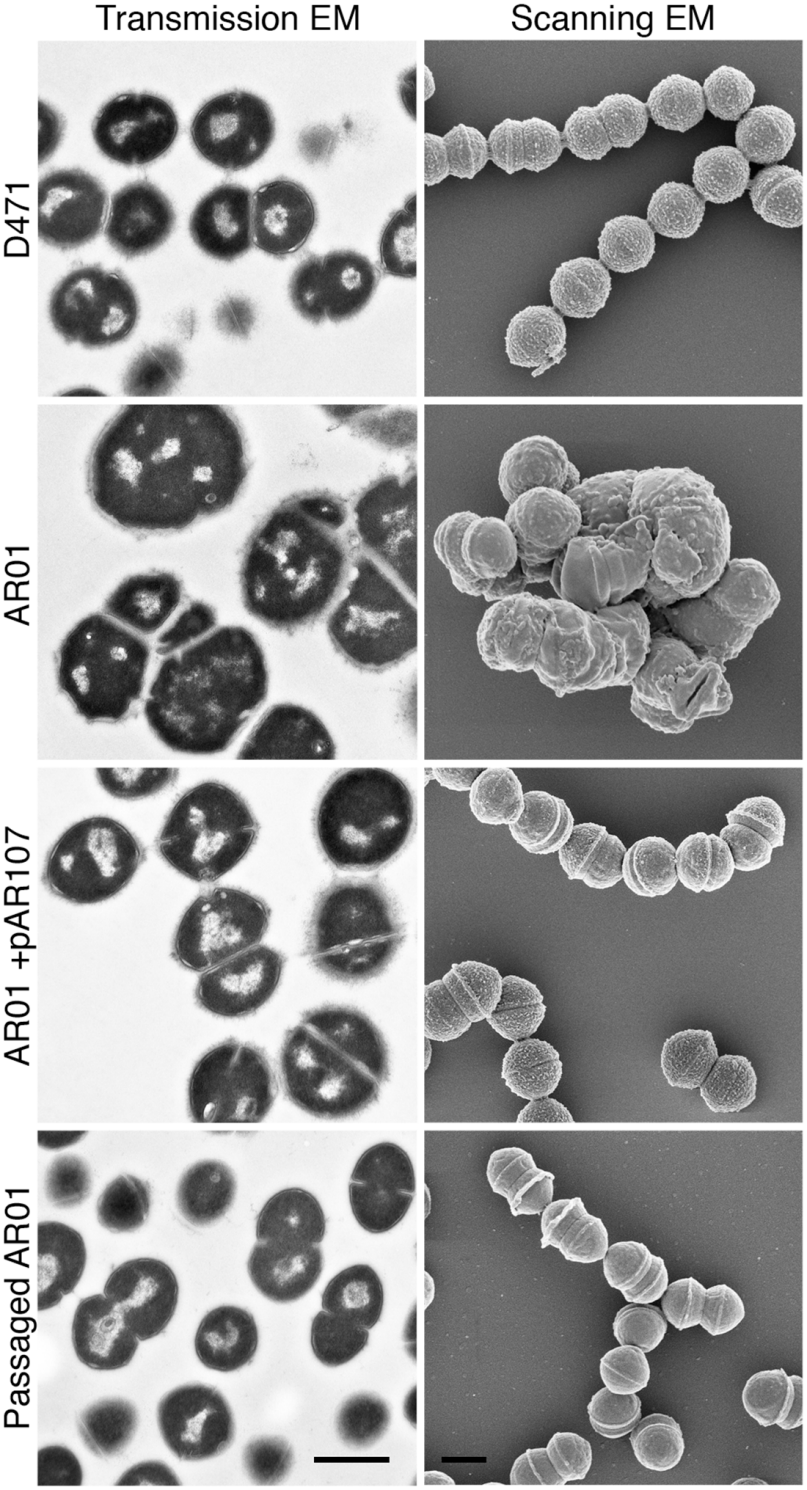
Deletion of *S*. *pyogenes* sortase results in aberrant cellular morphology. Wild type *S*. *pyogenes* D471, sortase mutant AR01, complemented AR01+pAR107, and a variant of AR01 that was passaged 6 times in TH+Y, were diluted from overnight cultures 1:100 into fresh media (containing spectinomycin for AR01+pAR107), grown to log phase, and fixed. The cells were processed for transmission and scanning electron microscopy as described in the Material and Methods section. Scale bars represent 500 nm.

To further characterize the nature of the morphological aberrations following deletion of sortase, the strains were examined by electron microscopy (Fig 1). As expected, the cells of wild type D471 were organized in chains and their surface was covered with M protein, seen as hair-like projections. Sortase mutant AR01 cells on the other hand, displayed morphological defects ranging from kinks or twists in the chains to enlarged cell volume and disruption of the septum, including incomplete septa and multiple septa per cell. M protein, which requires sortase for surface anchoring, was sometimes observed at the septal region of cells but did not uniformly cover the cell surface. Complementation of the mutant with the sortase expression plasmid pAR107 restored a near-wild-type morphology. Passage of the sortase mutant in laboratory medium resulted in a return to wild type chain organizaton, but the surface of the strain appeared smooth, and bare of proteins. Growth of the AR01 was slower than wild type D471 in TH+Y, but passaged strains regained normal growth kinetics (S1 Fig).

### Deletion of *S*. *pyogenes* sortase leads to accumulation of sorting intermediate with uncleaved LPXTG motifs

Accumulation of missorted surface proteins following deletion of sortase is one potential mechanism that could lead to the observed morphological aberrations. As discussed above, translocation of surface proteins across the membrane is stalled when the C-terminal hydrophobic sequence and positively charged residues reach the secretion channel. At the stalled state, sortase cleaves the LPXTG motif, and covalently attaches the protein to lipid II; the C-terminal protein fragment is released back into the cytoplasm [8, 10]. Deletion of sortase therefore prolongs the time surface proteins are found in a membrane-bound intermediate state, leading to the accumulation of sorting intermediates [2, 8, 15, 27, 32, 39].

To determine the extent and location of missorted surface proteins with an uncleaved LPXTG motif, we used a sortagging procedure. Sortagging uses recombinant sortase (typically *S*. *aureus* SrtA) to covalently attach a pentaglycine probe to a target protein, to which an LPXTG motif has been introduced [40]. In the sortase mutant, LPXTG motifs of native proteins are not processed, and could therefore be detected using sortagging. Log-phase cultures of D471, AR01, AR01+pAR107, and passaged AR01, were fixed, and attached to microscope slides. The cells were gently permeabilized with PlyC, using a procedure previously developed for immunofluorescence studies in this organism [39]. Permeabilization is required since the LPXTG motif of surface proteins is proximal to the membrane, beneath the cell wall, and is not accessible in intact cells. The permeabilized cells were blocked, and then incubated with a biotinylated pentaglycine probe in the presence or absence of recombinant *S*. *aureus* SrtA. Streptavidin-FITC conjugate was used to detect covalently bound biotin molecules. The combination of *S*. *aureus* sortase and a pentaglycine probe was chosen since it is the best-characterized and most robust form of sortagging. *S*. *aureus* sortase has been shown to recognize all variants of the LPXTG motif [41], and specifically, the cell wall sorting signal (CWS) of *S*. *pyogenes* M protein [7].

**Fig 2:**
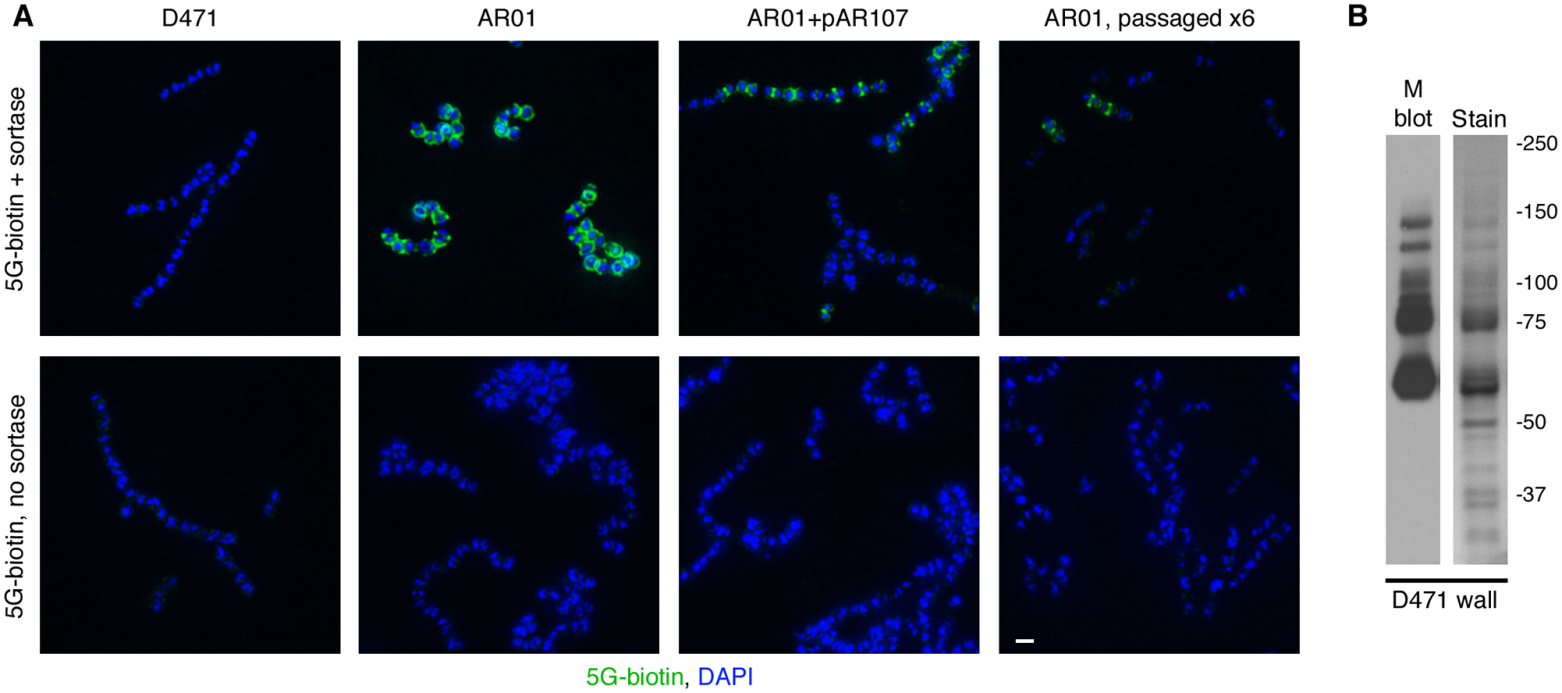
Missorted surface proteins accumulate in the sortase mutant AR01. A. Wild type *S*. *pyogenes* D471, sortase mutant AR01, complemented AR01+pAR107, and a passaged AR01 variant, were diluted from overnight cultures 1:100 into fresh media (containing spectinomycin for AR01+pAR107), grown to log phase, fixed, attached to glass cover slides, and permeabilized with PlyC. The cells were blocked and incubated with pentaglycine-biotin (5G-biotin) in the presence or absence of purified *S*. *aureus* sortase A. The slides were washed, and pentaglycine-biotins molecules (attached to LPXTG motifs by sortase) were detected using FITC-streptavidin. DAPI was used to visualize DNA. The scale bar represents 1 μm. Note that expression of sortase from pAR107 is lower than the wild type, explaining the presence of intact LPXTG motifs. B. The cell wall fraction of log-phase D471 cells (solubilized with PlyC in PBS 30% raffinose) was separated by SDS-PAGE. Duplicate gels were stained with GelCode blue or processed for Western blot using the 10B6 monoclonal antibody. The most prominent bands on the stained gel correspond to M protein.

Using this technique, no signal was detected in D471 cells, suggesting that there are relatively few unprocessed LPXTG motifs in wild type cells (Fig 2A), consistent with the rapid assembly speed observed in pulse-chase assays [8]. The sortase mutant AR01 on the other hand, displayed an intense fluorescent signal at the cells’ septa and circumference, indicating the presence of a large number of proteins with uncleaved LPXTG motifs. Complementation of the sortase mutant with pAR107, resulted in a drastic reduction in fluorescent signal. Interestingly, in some streptococcal chains, fluorescent signal was still observed, and was predominantly associated with septa and equatorial rings. Previous studies have shown that sortase production from pAR107 is roughly an order of magnitude lower than in wild type cells [39]. The observed fluorescent signal therefore, likely represents areas in which the amount of translocated surface protein exceeded the processing capacity of sortase in the cells. This may indicate that a larger number of molecules are translocated and anchored at the septum compared to other cellular regions. Finally, most passaged AR01 cells displayed no fluorescent signal, although a weak septal fluorescent signal was observed in some cells. Return of the sortase mutant to wild type chain organization following passage in TH+Y, was therefore accompanied by a substantial reduction in the presence of missorted surface proteins. This observation is in line with the theory that missorted surface proteins are the cause of the morphological aberrations.

In considering which surface proteins could be responsible for the observed morphological aberrations, it is meaningful that the fluorescent signal was primarily observed at the septum. Surface proteins in *S*. *pyogenes* are translocated through two distinct pathways either at the division septum or periphery [16, 17]. Anchoring through the septal pathway is rapid and efficient, and occurs at a very narrow septal band, compared to the gradual accumulation of proteins on large cellular areas through the peripheral anchoring pathway [17].

The presence of a YSIRK G/S motif in the signal sequence of Gram-positive surface proteins is correlated with translocation at the septum [16, 26]. We analyzed the published genome sequence of the M6 strain MGAS10394 for the presence of proteins containing a CWS motif (See S1 Materials and Methods for details, alignment of the resulting CWS is presented in S2 Fig). We further analyzed the signal sequence of the resulting proteins for the presence of a YSIRK G/S motif (S3 Fig). We found that as opposed to results reported in *S*. *aureus*, where 13 of 20 surface proteins have a highly conserved YSIRK G/S motif in their signal sequence [26], only two of the 16 identified MGAS10394 surface proteins contain an intact YSIRK G/S motif (M protein and ScpC), while two additional proteins have a partial YSIRK G/S motif (GRAB, PulA), and one has a substantially altered motif (5’-nucleotidase). We also found that the distance between the YSIRK G/S motif and the predicted signal peptidase cleavage site in *S*. *pyogenes* varies by up to eight amino acids, as opposed to a maximum variation of two amino acids in *S*. *aureus*. All other *S*. *pyogenes* surface proteins lack a YSIRK G/S motif in their signal sequence. The YSIRK G/S motif is therefore both less common and less conserved in *S*. *pyogenes* compared to *S*. *aureus* (S3 Fig).

Of all the proteins analyzed, M protein has the most conserved YSIRK G/S motif, and is the only protein experimentally shown to be anchored at the septum [16, 17, 24, 42]. M protein is a coiled-coil molecule that is clearly visible in electron micrographs as a dense meshwork of hair-like structures on the surface of *S*. *pyogenes* cells [43]. To determine how abundant M protein is on the wall of *S*. *pyogenes* D471, we analyzed cell wall fragments of this strain (released by PlyC digestion in PBS in the presence of 30% raffinose to prevent osmotic lysis) by SDS-PAGE and Western blot using an M protein-specific monoclonal. This revealed that the major protein bands visible on a stained gel correspond to M protein (Fig 2B). The ladder pattern observed following lysin digestion of the cell wall results from attachment of M protein to cell wall fragments of different sizes, a phenomenon routinely observed for M protein and other surface proteins [17, 39, 44–47]. M protein is therefore by far the most abundant surface protein in log-phase cells. This observation suggests that M protein likely accounts for the majority of the observed signal in the sortagging assay, whereas weakly expressed surface proteins may contribute little or may even be below the detection threshold. It also indicates that missorting of M protein likely contributes significantly to the aberrant morphology of the sortase mutant.

### Passaged sortase mutants with normal morphology have a reduced level of M protein

Given the large number of M protein molecules missorted at the septum of the sortase mutant, we tested whether a return of mutant to normal morphology following passage in laboratory medium was accompanied by a reduction in M protein expression level. We also tested in parallel the expression level of the less abundant, peripherally anchored SfbI. An aliquot of wild type D471, sortase knockout AR01, and complemented AR01+pAR107, were sonicated to separate the chains into mostly single cells and occasionally diplococci (validated by microscopy), and streaked on TH+Y plates (containing spectinomycin for AR01+pAR107). This procedure was performed to ensure that colonies represent the progeny of a genetically homogeneous organism. Colonies were picked into liquid medium and passaged six times as described above. A sample of each overnight culture was separated into supernatant and pellet, and these were stored at -80°C until use; glycerol stocks were prepared for cultures from the first and last days of the experiment.

**Fig 3:**
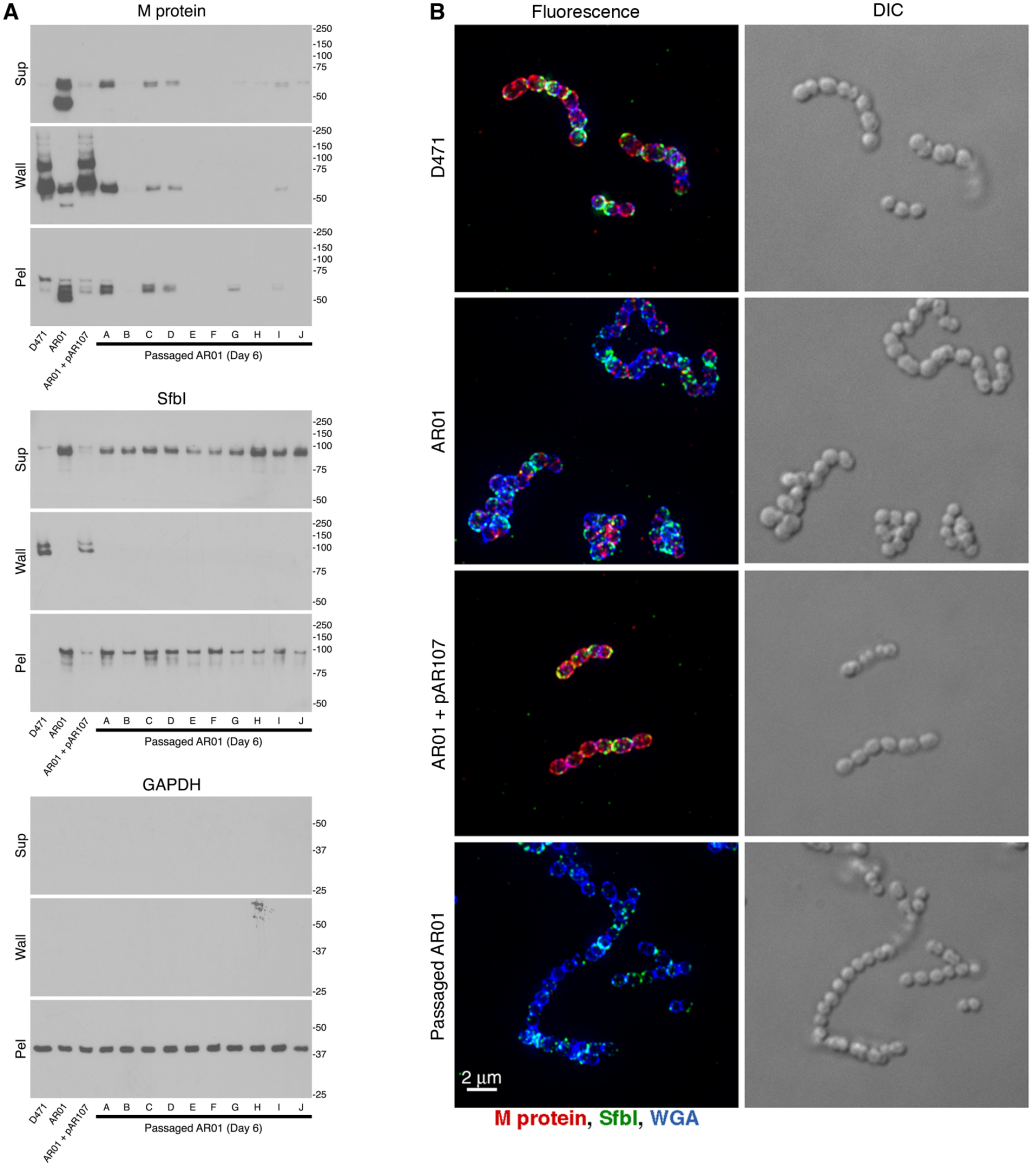
Passage of the sortase mutant AR01 in TH+Y results in loss of M protein expression. **A** Wild type *S*. *pyogenes* D471, sortase mutant AR01, complemented AR01+pAR107, and 10 separate variants of AR01 that were passaged 6 times in TH+Y, were diluted from an overnight culture 1:100 into fresh media (containing spectinomycin for AR01+pAR107), grown to log phase, fractionated into supernatant, wall (solubilized with PlyC in PBS 30% raffinose), and spheroplast pellet, and examined by Western blot. The monoclonal antibody 10B6 was used to detect M protein, specific serum was used to detect SfbI, and the monoclonal antibody 3A1 was used to detect cytoplasmic glyceraldehyde 3-phosphate dehydrogenase (GAPDH), as loading control. **B** Cells grown in a similar manner were fixed, and processed for fluorescence microscopy as described in the Materials and Methods section. Specific antibodies were used to label M protein (red) and SfbI (green). The cell wall was stained with WGA marina blue (blue). Images were obtained using deconvolution immunofluorescence and Nomarski microscopy. Deconvolution images are presented as maximum intensity projections, composed of all the Z-sections. Additional images are presented in S4 Fig.

The amount and distribution of M protein and SfbI were first analyzed by Western blot. Log phase D471, original AR01, AR01+pAR107, and 10 different 6-day-passaged variants of AR01, were diluted 1:100 from an overnight culture, grown to log phase, and fractionated into supernatant, cell wall (solubilized with PlyC in PBS 30% raffinose), and spheroplast pellet. As expected, M protein and SfbI were found mostly in the wall fraction of wild type D471 and complemented AR01+pAR107, but were missorted to the supernatant, wall, and spheroplast fractions of the sortase mutant AR01 (Fig 3A). However, the amount of M protein was clearly reduced in all the passaged AR01 strains, although not to the same extent. SfbI level was not substantially reduced in the passaged strains. Cytoplasmic GAPDH was used as loading control.

We also examined the morphology of the strains, and the distribution of surface-exposed M protein and SfbI by microscopy (Fig 3B). Immunofluorescence images showed that M protein (red) and SfbI (green) had a typical distribution on the surface of wild type D471, and complemented AR01+pAR107, whereas patches of non-covalently bound proteins were observed on the surface of the sortase mutant AR01. Consistent with the Western blot results, M protein fluorescence on the passaged AR01 variants was very low. SfbI fluorescence was not as dramatically affected, and was still visible on the cells. Additional phase-contrast and fluorescence microscopy images of 10 passaged variants, as well as additional controls, can be found in S4 Fig.

**Fig 4:**
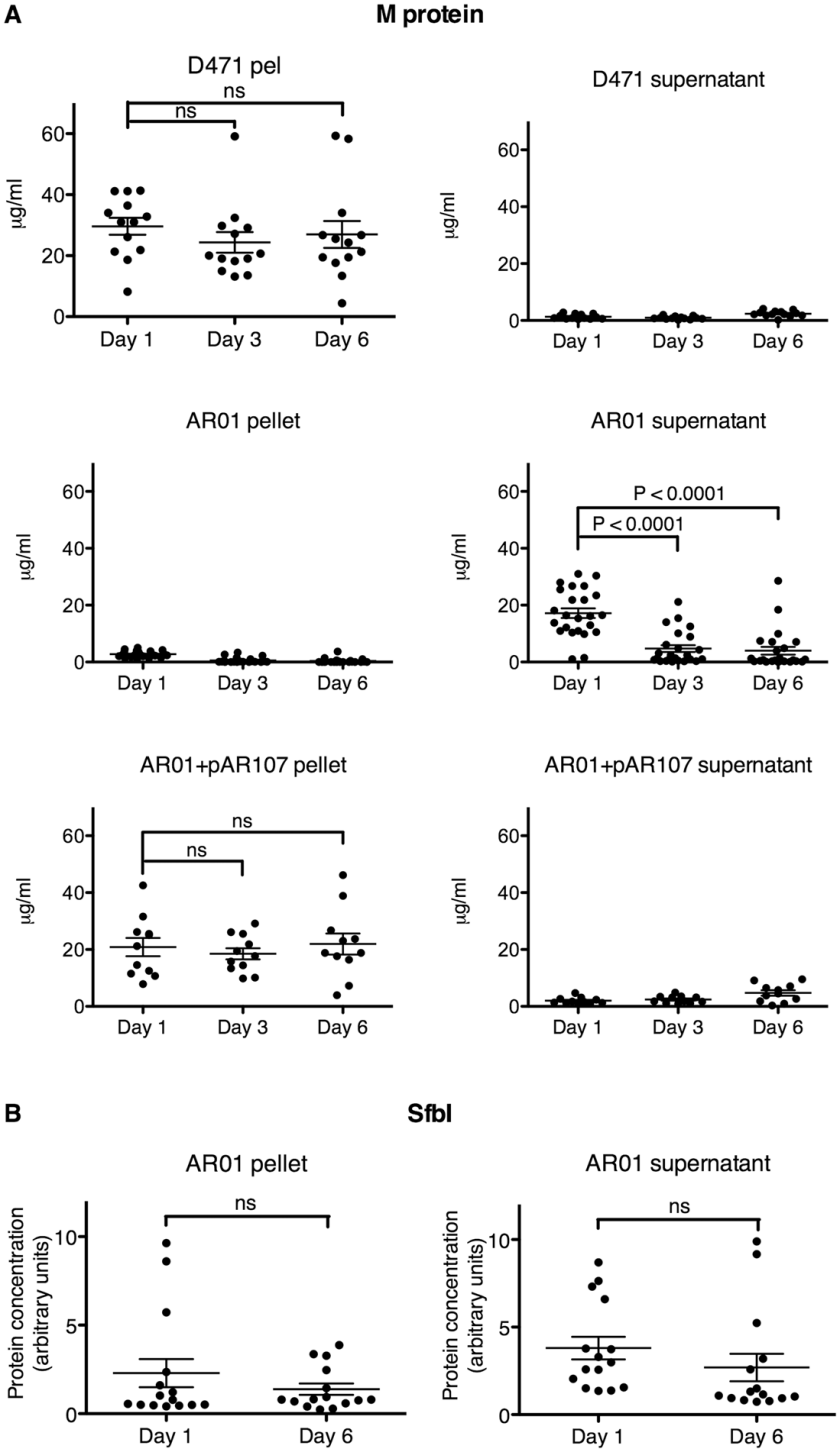
The *S*. *pyogenes* sortase mutant is under selective pressure leading to the loss of M protein expression. **A** Wild type D471, sortase mutant AR01 (original stock), and complemented AR01+pAR107, were sonicated to separate the streptococcal chains into single cells, and subsequently plated. Single colonies were picked and grown in TH+Y (containing spectinomycin for AR01+pAR107) at 37°C overnight. Each day, a seed culture was transferred to a new tube for overnight growth, and a sample of the original culture was separated into supernatant and cell pellet, and stored at -80°C until use; the cultures were passaged in this manner six times. The cell pellets were lysed with PlyC. M protein was quantified by capture ELISA as described in the Materials and Methods section. The results represent 13 repeats for D471, 24 repeats for AR01, and 11 repeats for AR01+pAR107. **B** The relative amount of SfbI in AR01 supernatant and cell lysate was similarly determined by capture ELISA; the results represent 15 repeats. See S6 Fig for *sfbI*-negative control.

The amount of M protein and SfbI in cultures from different stages of the passage experiment was also quantified by ELISA. For this purpose, the passage experiment was repeated three more times, and the cell pellets (lysed with PlyC) and culture supernatants were collected each day. Samples from 25 AR01 colonies, 11 AR01+pAR107 colonies, and 13 D471 colonies, were collected at days 1, 3, and 6, and analyzed by capture ELISA. As expected, the majority of M protein was found in the supernatant of the sortase mutant AR01, but in the cell pellet of wild type and sortase complemented strains. A substantial amount of M protein was still detected in most AR01 cultures during the first day’s passage. By the third day, and even more so by the sixth day, M protein expression level was drastically reduced (Fig 4A). Interestingly, not all colonies lost M protein expression at the same time, with a few colonies losing M protein expression as early as the first day, and conversely, a few colonies maintaining normal M protein expression even at the sixth day. This points to a stochastic model, where variants expressing less M protein arise in the population at different times and eventually take over. On the other hand, M protein level did not decrease in wild type D471 or complemented AR01+pAR107. Similar results were obtained with a semi-quantitative dot blot assay, which also included GAPDH as loading control (S5 Fig). These results indicate that M protein expression is under a negative selective pressure in the sortase mutant, further supporting a role for M protein missorting as a major cause for the observed morphological aberrations in the mutant strain. This report focuses on understanding the implications of protein missorting in the *S*. *pyogenes* sortase mutant; the genetic mechanisms leading to reduction in M protein expression will be adressed elsewhere.

The amount of peripherally-anchored SfbI was analyzed at days 1 and 6 in a subset of 15 AR01 cultures, selected at random from the different passage experiments. While a slight reduction in SfbI level was observed in some of the colonies, as a whole this difference was not statistically significant (Fig 4B). *S*. *pyogenes* SF370, which does not contain an *sfbI* gene [48], showed no signal in this assay, confirming its specificity (S6 Fig).

### Deletion of sortase in a strain lacking M protein does not result in morphological aberrations

**Fig 5:**
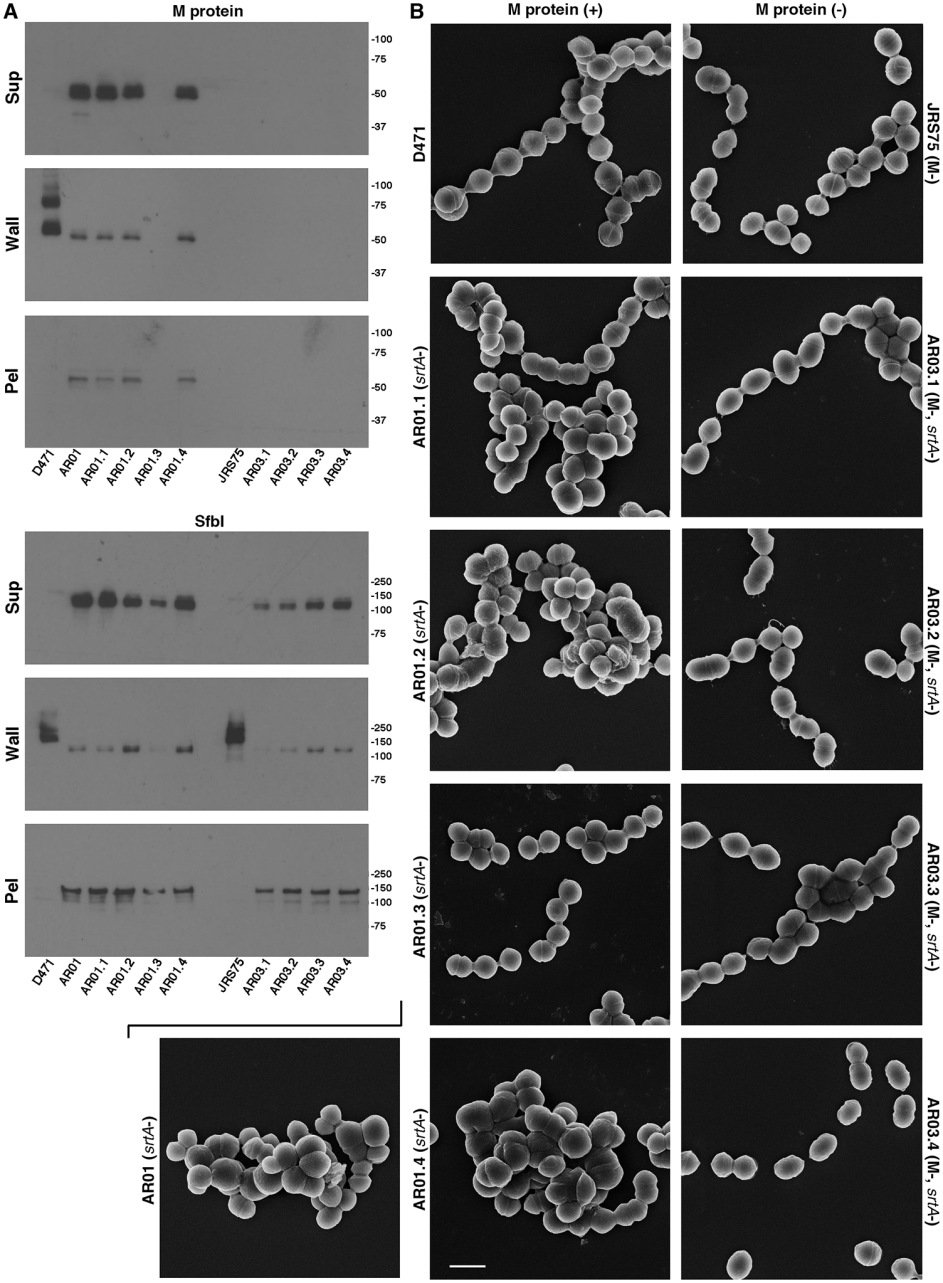
Deletion of sortase in a strain lacking M protein does not lead to morphological aberrations. **A** Wild type D471, D471-derived sortase mutants (original AR01, and new AR01.1-AR01.4), M-negative JRS75, and JRS75-derived sortase mutants (AR03.1-AR03.4), were each diluted from an overnight culture 1:100 into fresh TH+Y and grown to log phase. Cultures were fractionated into supernatant, wall (solubilized with PlyC in PBS 30% raffinose), and spheroplast pellet, and examined by Western blot. The monoclonal antibody 10B6 was used to detect M protein, and specific serum was used to detect SfbI.**B** Cells grown in a similar manner were fixed and examined by scanning electron microscopy as described in the Materials and Methods section; the scale bar represents 1 μm. Note that while one of the D471-derived sortase mutants (AR01.3) did not display morphological aberrations, this strain had lost the expression of M protein early, as observed by Western blot.

To further examine the correlation between surface protein missorting and morphological aberrations, we deleted sortase from the M protein mutant strain JRS75. Due to the high abundance of M protein, its deletion represents a substantial reduction in the overall load of surface proteins in the cells. Four mutants with the deletion of both M protein and sortase genes were isolated, and designated AR03.1-AR03.4. We also created four additional D471-derived, M-protein-positive sortase-negative mutants (designated AR01.1-AR01.4), in order to establish how reproducibly mutants with altered morphology could be isolated, given the observed unstable nature of this phenotype during growth in TH+Y. Following analysis of gene deletion by PCR, deletion of sortase was functionally validated by examining the anchoring patterns of M protein and SfbI by Western blot (Fig 5A). Surface proteins in these strains were missorted to the supernatant, wall, and pellet fractions, as is expected for a sortase mutant; no M protein was detected in JRS75 and its sortase mutants. One of the four new D471-derived sortase-deletion-only mutants (AR01.3) had a reduced level of M protein expression, which we interpret as an early selection of a low-M variant during the isolation of this sortase mutant. When examined by scanning electron microscopy, many cells of the three other sortase-negative strains that retained M protein expression (AR01.1, AR01.2, AR01.4) displayed morphological aberrations resembling the original AR01 strain, while AR01.3, which lost M protein expression early, displayed normal chain organization (Fig 5B). This is another case therefore, where a connection was observed between a reduction in M protein expression level, and a return to normal chain organization. In contrast to the sortase deletion mutants derived from wild type D471, none of the M protein and sortase double mutants displayed morphological aberrations (Fig 5B). This further supports the hypothesis that missorted surface proteins at the septum cause the observed morphological aberrations. To determine whether the new D471-derived sortase mutants were also under selective pressure for a reduction in M protein expression level, these strains were subjected to a small-scale passage experiment (two colonies each, analyzed by dot-blot, with two repeats of the experiment), which yielded results similar to original AR01. AR01.3 maintained its initial low level of M protein throughout this experiment (S7 Fig).

### Deletion of sortase leads to an increase in membrane permeability, however normal membrane integrity is restored following passage in TH+Y

**Fig 6:**
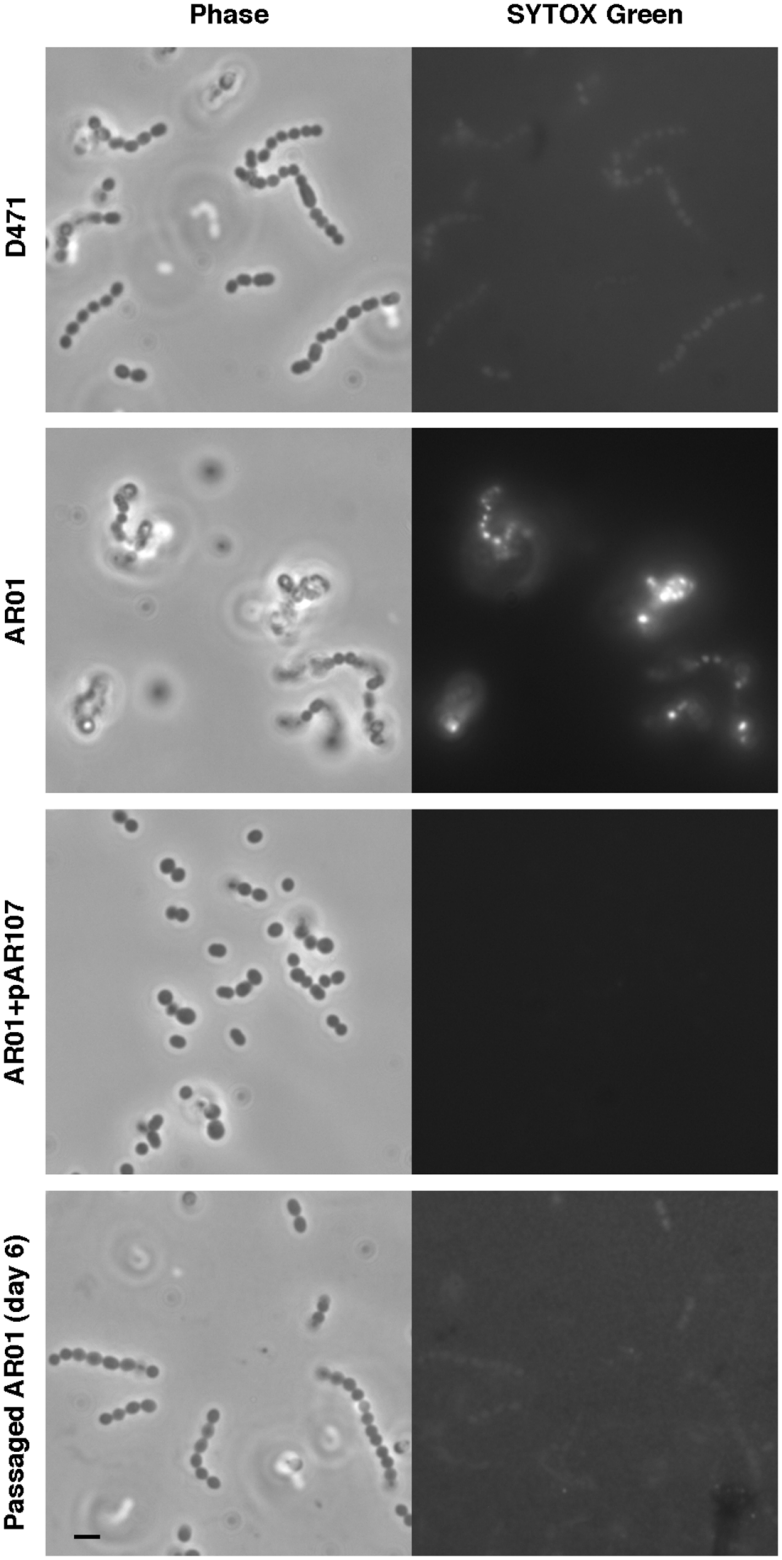
The sortase mutant, but not the passaged strain, displays increased membrane permeability. Wild type D471, sortase mutant AR01 (original stock), complemented AR01+pAR107, and a low-M passaged variant of AR01, were diluted 1:50 from an overnight culture and grown to OD_600_ 0.5. The cells were incubated with 5 mM SYTOX green for 30 min at room temperature, washed with PBS, and immediately imaged. The scale bar represents 2 μm. Additional images are presented in S8 Fig.

Accumulation of sorting intermediates in the membrane could potentially compromise its integrity. To test this, wild type D471, sortase mutant AR01, complemented AR01+pAR107, and a passaged low-M variant of AR01, were grown to log phase, incubated with the membrane-impermeable fluorescent dye SYTOX green for 30 minutes, washed, and visualized by fluorescent microscopy. Original AR01 cells displayed a substantially stronger fluorescent signal than wild type D471 (although not as strong as that of dead cells), indicating that their membrane was compromised (Fig 6). No increase in membrane permeability was observed in sortase-complemented AR01+pAR107, or passaged AR01. The experiment was repeated three separate times, and additional data from all these experiments is presented in S8 Fig. The occurrence of increased membrane permeability in the original sortase mutant, but not the low-M passaged strain, suggests that missorted surface protein are likely to be a major cause for this phenotype.

### Deletion of sortase increases the sensitivity of *S*. *pyogenes* to human cathelicidin LL-37, however this effect is partially reversed in the low-M passaged mutant

Antimicrobial peptides (AMPs), such as cathelicidins and defensins, play an important role as a first line of defense against invading microbes [49–51]. Human cathelicidine LL-37 is important in the killing of phagocytosed microorganisms by immune cells, and is also produced by non-immune cells, primarily in barrier tissues. It functions by permeabilizing the bacterial membrane, and also has roles as an immunomodulator and chemoattractant [49, 52]. LL-37 is important in the clearance of *S*. *pyogenes*, and has a role in killing streptococci trapped in neutrophil extracellular traps (NETs) [53, 54].

**Fig 7:**
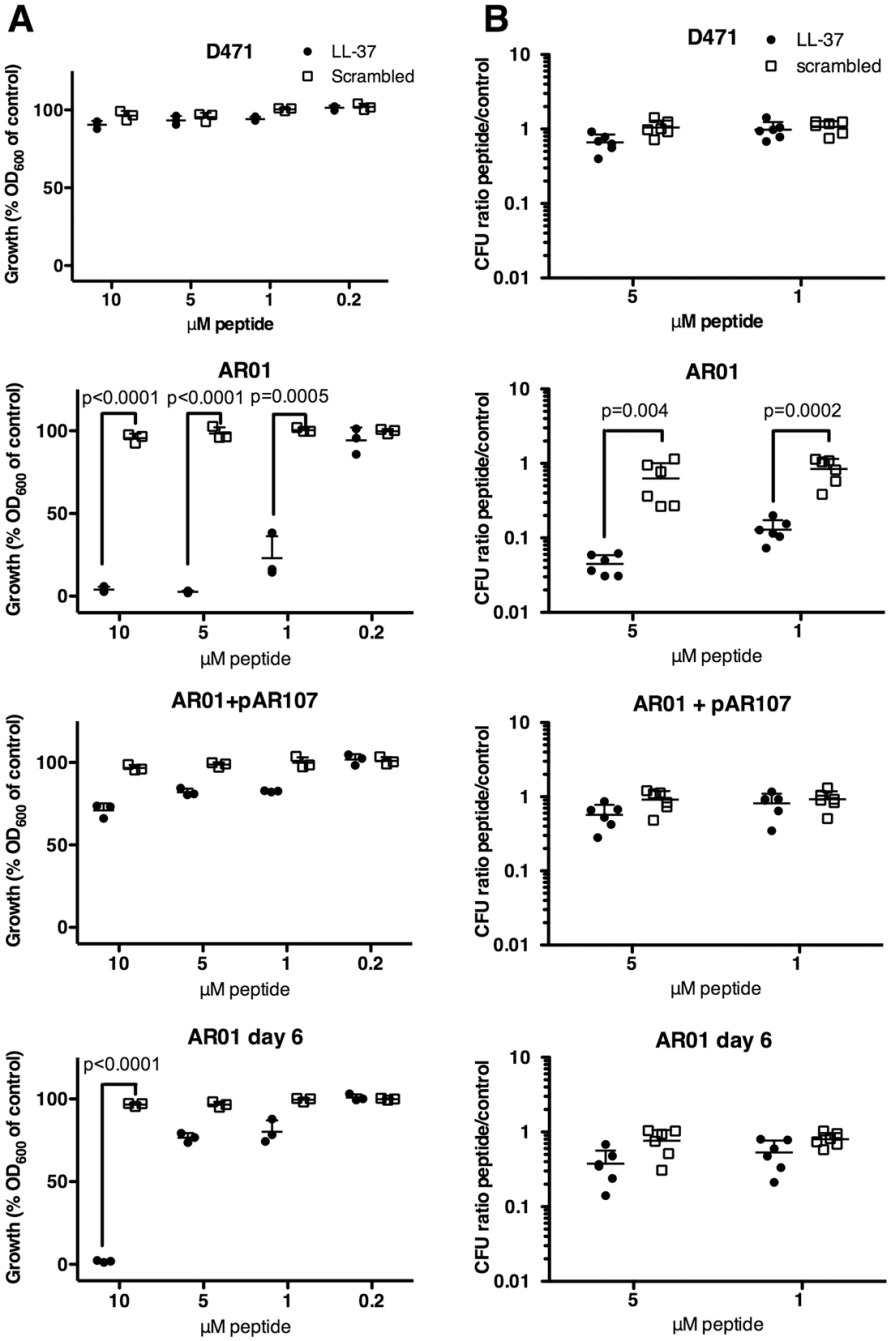
The sortase mutant is highly sensitive to LL-37. **A** Wild type D471, original sortase mutant AR01, sortase complemented AR01+pAR107, and passaged AR01 (day 6), were grown to OD_600_ 0.5, diluted 1:10^5^ in TH+Y (containing spectinomycin for AR01+pAR107) and mixed with LL-37, scrambled peptide, or TH+Y alone. The strains were incubated stationary in a 96-well plate for 16 h at 37°C, and the final OD_600_ was measured. The final OD_600_ value is presented as a fraction of the value obtained in the absence of peptides (defined as 100%). Experiments were repeated 3 times in triplicates, and a representative experiment is shown; error bars represent standard deviation. P-values for samples showing significant growth inhibition were calculated using t-test. **B** Cultures prepared in a similar manner were rotated in microfuge tubes for 3 h at 37°C, and then serially diluted and plated for CFU quantification. The CFU ratio treated/untreated is presented. Experiments were repeated 3 times in duplicates, and the results from all 3 experiments are shown; error bars represent standard deviation. P-values were calculated using t-test.

The altered cell wall structure and destabilized membrane of the sortase mutant suggest that it could be particularly sensitive to AMPs. To test this, we used a method modified from Sol *et al*. [55]. D471, AR01, AR01+pAR107, and passaged AR01, were grown to log phase, diluted 1:10^5^, and incubated in a 96-well plate for 16 hours at 37°C with varying concentrations of LL-37 or a scrambled peptide as control. A clear growth-inhibition was observed when the original AR01 strain was incubated in the presence of 10, 5, and 1 μM LL-37, but not with a control scrambled peptide, as determined by OD_600_ measurement (Fig 7A). Growth of the passaged strain was severely inhibited only at the 10 μM LL-37 concentration, with slight inhibition seen at lower concentrations. The growth of the wild type and the complemented sortase mutant was only slightly inhibited at the LL-37 concentrations tested.

We also used a CFU-based assay to test the effects of LL-37 on the different strains over a shorter time period. In this assay, log phase cells were diluted 1:10^5^ in TH+Y, rotated with different concentrations of peptides for 3 h at 37°C, and then serially diluted and plated for colony counts. LL-37 inhibited the growth of AR01 at the concentrations tested (5 μM, and 1 μM), but not the scrambled peptide (Fig 7B). The growth of other strains was only slightly inhibited at these concentrations, and this was not statistically significant.

One possibility is that surface proteins could play a role in LL-37 resistance, and that the increased sensitivity of the sortase mutant to LL-37 resulted from its inability to anchor surface proteins. Nevertheless, the absence of anchored surface proteins is not likely to account for the entirety of the phenotype observed in the original sortase mutant. This is because passaged sortase mutants, which similarly lack anchored surface proteins, are substantially less sensitive to LL-37. The much higher load of missorted surface proteins in the original sortase mutant, and the resulting altered cellular physiology, are likely to contribute substantially to the increase in LL-37 sensitivity. Passaged sortase mutants however, did not fully regain a wild type level of LL-37 resistance, suggesting that anchored surface proteins may also be important.

### *S*. *pyogenes* sortase mutant is susceptible to phagocytic killing; growth of the original sortase mutant, but not the passaged strain is severely inhibited in plasma

**Fig 8:**
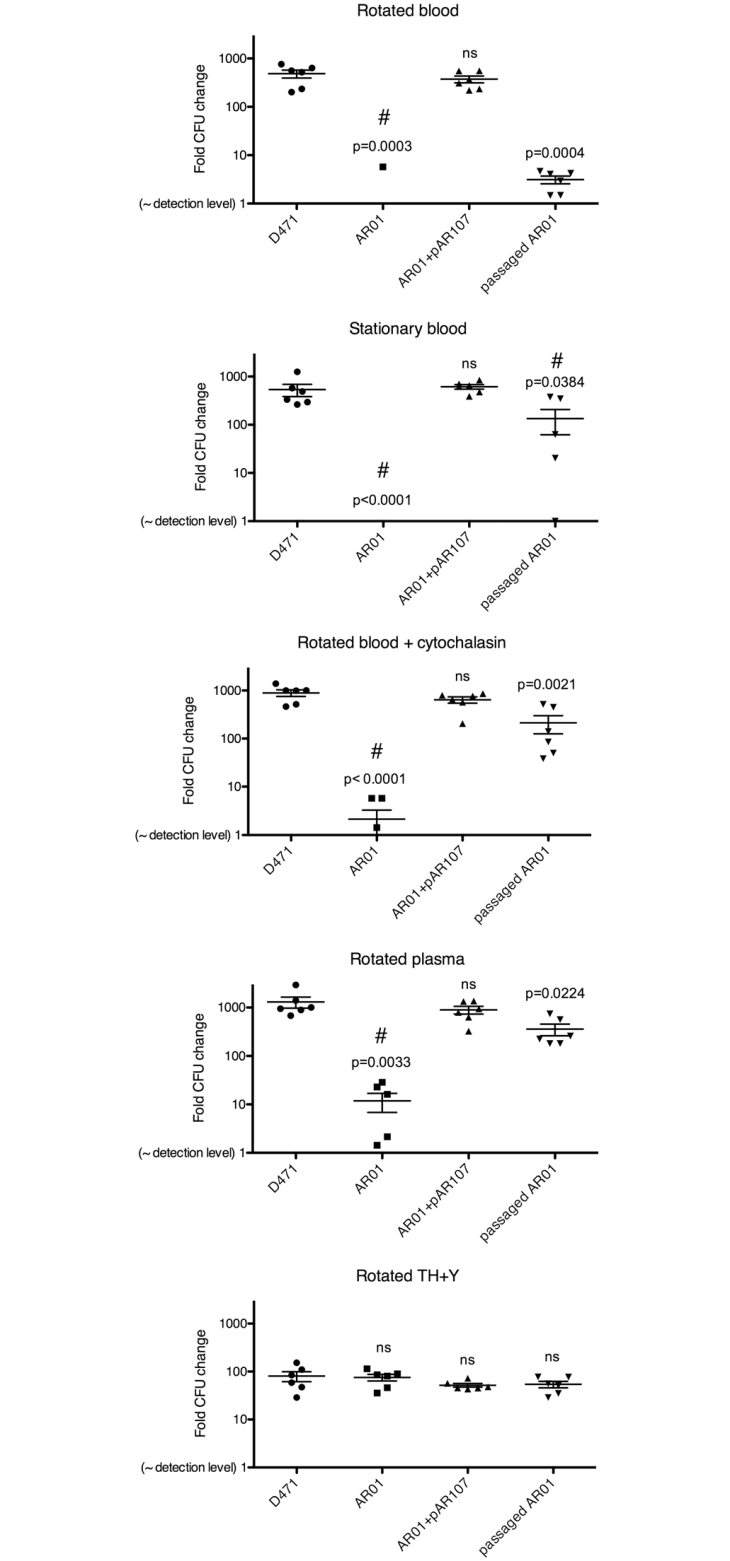
*pyogenes* sortase mutant is susceptible to phagocytosis in human blood, and grows poorly in plasma. ***S*.** Wild type D471, original sortase mutant AR01, sortase-complemented AR01+pAR107, and passaged AR01 (day 6), were grown to OD_600_ 0.2, diluted 1:10^5^, and incubated in the following conditions: 1) rotated in blood, 2) stationary in blood (phagocytes sink to the bottom), 3) rotated in blood + 20 μM cytochalasin B (inhibits phagocytosis), 4) rotated in plasma, 5) rotated in TH+Y. Blood and plasma were from a healthy donor who tested negative for antibodies specific to M protein serotype 6. Following 3 h incubation at 37°C, samples were serially diluted 10-fold, and plated on TH+Y for CFU quantification; results are presented as CFU_final_/CFU_start_. Experiments were repeated 3 times in duplicate, and the results from all 3 experiments are shown; error bars represent SEM. P values compared to wild type D471 were calculated using t-test; “ns” denotes no statistical significance. # symbol denotes that one or more data points in the group were below detection level.

M protein allows *S*. *pyogenes* to evade phagocytosis and is therefore essential for survival in human blood [18]. Deletion of sortase prevents the anchoring of M protein to the cell wall, making the mutant susceptible to phagocytosis. The original sortase mutant however, also displayed morphological aberrations, increased membrane permeability, and increased LL-37 sensitivity, which may also affect its survival in the blood environment. To test the effects of sortase deletion on survival in blood and plasma, wild type D471, original AR01, plasmid complemented AR01, and passaged AR01, were grown to log phase and incubated for 3 h at 37°C in human blood/plasma using conditions modified from Lancefield *et al*. [56]: 1) rotated in blood, where bacteria are in constant contact with phagocytes, 2) stationary in blood, in which phagocytes sink to the bottom of the tube, allowing bacteria to grow in the top portion, 3) rotated in blood containing 20 μM cytochalasin B, which inhibits actin polymerization and thereby prevents phagocytosis, 4) rotated in plasma, where no phagocytes are present, and 5) rotated in TH+Y as medium control. These conditions can be termed more generally as phagocytosis-competent blood (1), phagocytosis-deficient blood (2–4), and laboratory medium (5). Blood and plasma were collected from healthy volunteers that tested negative for antibodies specific to M protein serotype 6. Following incubation, samples were serially diluted and plated for CFU quantification (Fig 8).

As expected, wild type D471 grew as well in the phagocytosis-competent rotated blood as in the phagocytosis-deficient conditions (stationary, cytochalasin B, and plasma), demonstrating its ability to evade phagocytic killing. Growth in TH+Y was not as robust as in blood, suggesting that blood is a better growth environment for this organism. The original sortase mutant was eliminated in phagocytosis-competent (rotated) blood; this was predicted given the absence of anchored M protein from its surface. Surprisingly however, this strain also grew extremely poorly, or was completely eliminated, in all the phagocytosis-deficient conditions (stationary, cytochalasin B, and plasma). The poor survival of AR01 in these conditions was not due to a general growth defect since in TH+Y medium the difference between the sortase knockout and the wild type was much smaller. Complementation of the sortase mutant with the sortase expression plasmid, pAR107, restored the mutant’s ability to grow in both phagocytosis-competent and phagocytosis-deficient blood. The passaged sortase mutant grew very poorly in rotated blood, consistent with the absence of anchored of M protein from its surface. Contrary to the original sortase mutant however, this strain had only a slight growth defect in phagocytosis-deficient blood, growing >50× better than the original mutant. This observation suggests that the inability of the original sortase mutant to grow in phagocytosis-deficient blood was not entirely due to the absence of anchored surface proteins, as these were also absent from the passaged strain. The reduced survival in phagocytosis-deficient blood is therefore likely attributed to the deleterious effects of missorted protein on cellular physiology. These effects were much more significant in the blood environment compared to TH+Y. The absence of statistically significant difference between the AR01 and the other strains in TH+Y using CFU quantification, despite a clear difference in growth rate between the strains when measured by optical density (S1 Fig) can be explained by the lower sensitivity of the CFU method, which can only detect large differences (i.e. 10 or 100 fold).

This experiment was also repeated with M-negative JRS75 and its sortase mutant derivate AR03.1. As expected, both strains were efficiently eliminated in rotated blood, since in the absence of M protein *S*. *pyogenes* is exposed to opsonophagocytic killing (S9 Fig). In contrast to the result observed with AR01 however, only a slight reduction in growth was observed for AR03 in phagocytosis-deficient blood. Due to the absence of M protein, the overall load of missorted surface proteins at the septum of AR03 is much lower than that of AR01, and accordingly this strain displayed no morphological aberrations (Fig 5B). In both morphology and survival in the blood environment therefore, AR03 resembled a passaged low-M variant of AR01. In combination, these results show a clear correlation between accumulation of sorting intermediates (and not merely the absence of sortase) and a reduction in fitness in the host environment through a phagocytosis-independent mechanism.

### Missorting of M protein with an altered LPXTG motif causes a reduction in fitness in the host environment that resembles the phenotype seen in the original sortase mutant

To directly test the effects of missorted surface protein in the host environment, we created three different M protein expression plasmids, where M protein contains either an intact LPXTG motif (LPSTGE), a reverse motif (EGTSPL), or a scrambled motif (TEPGSL). Altered LPXTG motifs are not recognized by sortase and therefore lead to missorting of the protein. These plasmids, as well as the parental empty plasmid pAR161, were transformed into wild type D471 cells. This makes it possible to specifically study the effects of M protein missorting in a strain that has sortase and all native surface molecules (including M protein).

**Fig 9:**
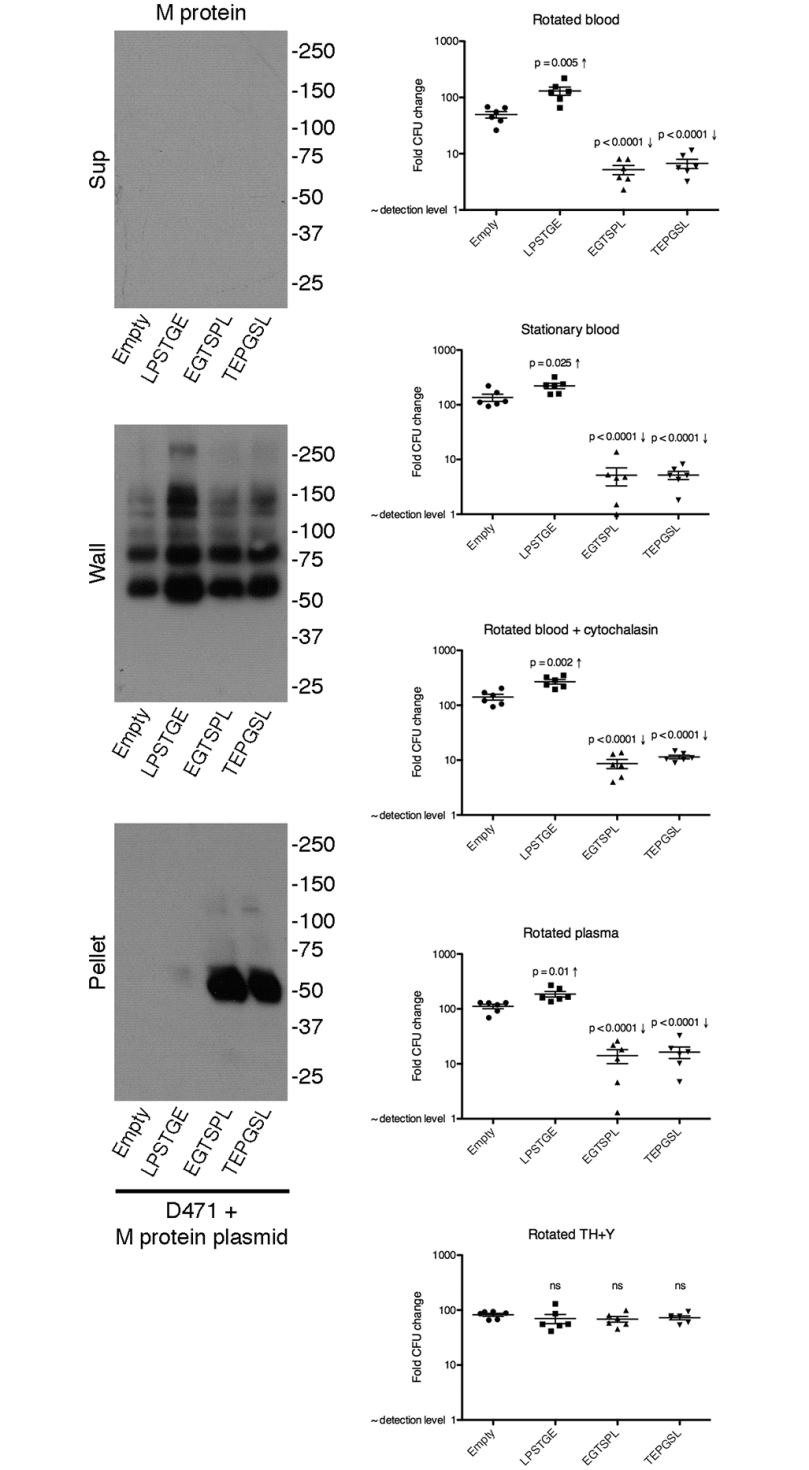
Expression of M protein with altered LPXTG motif in wild type D471 reduces fitness in the host environment. **A** D471 containing plasmids pAR161 (empty), pAR186 (LPSTGE), pAR184 (EGTSPL), or pAR185 (TEPGSL), was diluted from an overnight culture 1:100 into fresh TH+Y containing spectinomycin, grown to log phase, and fractionated to supernatant, cell wall, and spheroplast fraction pellet fractions. Fractions were analyzed by Western blot, and M protein was detected using the monoclonal 10B6. **B** The four strains were grown to OD_600_ 0.2, diluted 1:10^5^, and incubated in the following conditions: 1) rotated in blood, 2) stationary in blood (phagocytes sink to the bottom), 3) rotated in blood + 20 μM cytochalasin B (inhibits phagocytosis), 4) rotated in plasma, 5) rotated in TH+Y. Blood and plasma were from a healthy donor who tested negative for antibodies specific to M protein serotype 6. Following 3 h incubation at 37°C, samples were serially diluted 10-fold, and plated on TH+Y for CFU quantification; results are presented as CFU_final_/CFU_start_. Experiments were repeated 3 times in duplicate, and the results from all 3 experiments are shown; error bars represent SEM. P values compared to wild type D471 were calculated using t-test; “ns” denotes no statistical significance.

Overnight cultures of the four strains were diluted 1:100 into fresh media, grown to log phase and fractionated into supernatant, wall, and pellet fractions; the distribution of M protein in the different fractions was examined by Western blot (Fig 9A). Since the parental D471 strain expresses M protein from the genomic locus, all the derivative strains are expected to possess anchored M protein in the cell wall fraction. The native distribution of M protein expressed from the genomic location is observed in D471 + empty vector, where the majority of M protein is found in the wall fraction. Expression of M protein with an intact LPXTG motif from a plasmid increased the amount of M protein in the wall fraction, indicating that it was properly anchored to the cell wall by sortase. Conversely, expression of M protein with a reverse or scrambled LPXTG motif resulted in accumulation of the protein in the pellet fraction, indicating that it was not processed by sortase. Expression of M protein with a reverse or scrambled LPXTG motif did not disturb the anchoring of native M protein from the genomic locus, since the amount of M protein found in the wall fraction was similar to that found in the strain complemented with an empty plasmid. Since all the strains used here contain at least the wild type level of M protein anchored to their cell wall, any difference in their survival cannot be attributed to the absence of anchored M protein.

We repeated the blood rotation experiment using the four strains described above. As expected, D471 + empty vector grew well in both rotated blood and phagocytosis-deficient blood (stationary, cytochalasin B, and plasma). Expression of native M protein (LPSTGE) from a plasmid increased the fitness of D471 in rotated blood, and to some extent also in phagocytosis-deficient blood. This also is not surprising given the importance of M protein in the host environment, particularly in preventing phagocytosis. Expression of M protein with a reverse or scrambled LPXTG motif on the other hand, caused a dramatic reduction in fitness in both phagocytosis-competent, and phagocytosis-deficient blood. These effects resembled those observed in the sortase mutant. Reduction in fitness was specific to the host environment and was not seen in TH+Y. These results demonstrate that accumulated sorting intermediates directly lead to a reduction in fitness in the host environment, even in the absence of phagocytosis.

## Discussion

In this work we showed that accumulation of missorted surface proteins in the *S*. *pyogenes* sortase mutant has profound effects on cellular morphology and fitness in the host environment. Accumulation of sorting intermediates in the membrane of sortase mutants is well-documented at the biochemical level [2, 8, 27, 32]. During translocation across the membrane, the hydrophobic region and positively charged residues in the CWS prevent release of surface proteins into the surrounding medium. This stall in translocation, while essential for surface anchoring by sortase, leads to accumulation of sorting intermediates when sortase is absent.

Here we expanded on this knowledge by using a sortagging assay for the first time to examine the cellular location of stalled sorting intermediates in a sortase mutant. Wild type streptococcal cells contained relatively few proteins with an uncleaved LPXTG motif, indicating that sortase efficiently processes surface proteins soon after they are translocated across the membrane, consistent with biochemical data [8]. On the other hand, the sortase mutant had a large quantity of missorted surface proteins with uncleaved LPXTG motif. Of particular interest was the distribution of sorting intermediates under suboptimal sortase concentration, which showed intense signal primarily at the septa and equatorial rings, suggesting that on a molar basis, most molecules are anchored at these cellular locations. This is in line with the observed distribution pattern of lipid II (to which surface proteins are anchored) and sortase foci to these locations [39].

### Accumulation of missorted proteins leads to morphological aberrations

Surface proteins have not evolved to remain tethered to the membrane in a stable fashion. These proteins have a large surface exposed N-terminal region, followed by a proline-rich region that traverses the cell wall peptidoglycan, and a CWS at the C-terminus, which at least initially is embedded in the membrane. When the CWS is not processed, a large number of surface molecules may be attached to both the cell wall and membrane, possibly restricting membrane fluidity. This is the likely reason for the increased membrane permeability of the sortase mutant, observed using SYTOX green. Stalled surface proteins could also overload the secretion channels, potentially delaying the translocation of important secreted and membrane-bound factors. The septum is a highly dynamic cellular region and is the only site of cell wall assembly in *S*. *pyogenes* [24, 57]. Accumulation of sorting intermediates at the septum may therefore be particularly harmful. This could explain the morphological aberrations observed in the sortase mutant, including incomplete and misplaced septa, as well as multiple septa per cell.

Several lines of evidence suggest that the phenotype observed in the sortase mutant is caused directly by missorted surface proteins. First, accumulation of sorting intermediates was indeed detected in the sortase mutant using a sortagging assay, while no such intermediates were detected in the wild type strain. Secondly there was a strong selective pressure against the expression of surface proteins in general (as indicated by the sortagging assay), and M protein in particular, in the sortase mutant. In all cases observed, there was a direct correlation between a reduction in M protein expression and a return of the sortase mutant to wild type morphology. Also, when additional sortase mutants were isolated, all the mutants that retained M protein expression displayed morphological aberrations, while the single mutant that lost M protein expression early had no morphological defects. Selective pressure against M protein expression in the sortase mutant indicates that missorting of this protein is deleterious. Third, deletion of sortase from a strain lacking M protein did not result in morphological aberrations, again showing that missorting M protein in particular causes the morphological aberrations, and not a non-specific effect of sortase deletion. Finally, expression of M protein with a reverse or scrambled LPXTG motif in a wild type strain mimicked the effects seen in the original sortase mutant in the host environment. Despite the strong correlation between M protein missorting at the septum and morphological aberration, it is possible that other proteins that are also missorted at the septum, contribute to the overall effect.

Recently, Le Breton et al. screened a highly saturated transposon library of *S*. *pyogenes* strains 5448 (serotype M1T1) and NZ131 (serotype M49) to identify essential genes [58]. Interestingly, sortase A was found to be essential or critical (defined as a gene whose insertional inactivation was highly underrepresented in the library screen) in these strains. When we analyzed the genomes of these strains for proteins containing a cell wall sorting signal as described above, none corresponded to an essential gene in Le Breton’s data set. These results are therefore in agreement with our proposed model, whereby reduced viability of a sortase mutant can result from accumulation of sorting intermediates, even in environments where anchored surface proteins are not required.

### Effects of sortase deletion in the host environment

It is evident that even in laboratory medium, accumulation of surface intermediates in the sortase mutant led to distinct deleterious effects in the form of aberrant morphology, reduced growth rate, and increased membrane permeability. Nevertheless these effects were substantially exasperated in the presence of the antimicrobial peptide LL-37 and in the host environment. The AMP cathelicidine LL-37 is found in many human tissues, and is particularly abundant at the site of streptococcal skin and soft tissue infection [59]. The original sortase mutant was highly susceptible to LL-37, while passaged strains were less susceptible, but did not regain a wild type level of resistance. M protein of certain serotypes (for example M1 but not M49) confers LL-37 resistance through direct binding of the peptide [54]; no data is available for M6 protein. Since deletion of sortase prevents the anchoring of surface proteins, absence of surface proteins such as M protein could be one reason for the increased sensitivity. Nevertheless, the original sortase mutant was dramatically more sensitive to LL-37 than the passaged strain even though both strains similarly lack anchored surface protein, indicating that additional mechanisms are at play. Perturbation of the membrane by LL-37 is an important mechanism leading to killing of bacteria [51, 60]. The presence of a large quantity of missorted surface proteins in the membrane of the original sortase mutant, which was shown to compromise its integrity using SYTOX green, is the likely reason for this increase in LL-37 sensitivity. The aberrant wall morphology could also play a role, by allowing LL-37 easier access to the membrane.

The reduced fitness of the sortase mutant in blood and plasma could similarly result from the absence of anchored surface proteins, or the deleterious effects of missorted proteins. We distinguished between the phenotype observed in rotated blood, where opsonophagocytic killing by neutrophils is a major mechanism for the control of *S*. *pyogenes* growth, and blood whose cellular components have been removed or otherwise neutralized, leaving only plasma components to control bacterial growth (“phagocytosis-deficient blood”). Surface-anchored M protein allows *S*. *pyogenes* to evade phagocytic killing in the blood environment [18], and accordingly, wild type *S*. *pyogenes* grew well in both phagocytosis-competent and phagocytosis-deficient blood. The absence of anchored M protein from the wall of both original and passaged sortase mutants prevented their survival in phagocytosis-competent blood. It is in phagocytosis-deficient blood where the deleterious effects that resulted specifically from missorted surface proteins were most apparent. The original sortase mutant had a very low fitness in all the phagocytosis-deficient blood conditions, while low-M passaged sortase mutants, which did not suffer from the toxic effects of missorted surface proteins, grew nearly as well as the wild type. This indicates that the low fitness of the original sortase mutant in phagocytosis-deficient blood was not entirely due to the absence of anchored surface proteins since both the original and the passaged sortase mutants similarly lack those. The deleterious effects of missorted surface proteins in the original sortase mutant are therefore the most likely explanation. One or more of the AMPs found in plasma [50–52] are likely to play a role, given the observed sensitivity of the original sortase mutant to cathelicidin LL-37.

The deleterious effects of missorted surface proteins on survival in the host environment were further demonstrated by artificially inducing protein-missorting stress in wild type cells. Expression of an additional copy of M protein with an altered LPXTG motif, not recognized by sortase, led to a reduction in fitness in the host environment, in a manner similar to that observed in the sortase mutant. This is despite the presence of native M protein on the cell wall of the organism, expressed from the genomic copy. Missorted surface proteins therefore directly compromise fitness in the host.

### Surface protein expression in sortase mutants

M protein is among the most important virulence factors of *S*. *pyogenes* [19]. An interesting finding was that M protein level was reduced reproducibly following sequential growth of the sortase mutant in laboratory medium. This process was stochastic, suggesting that variants with a lower M protein level arose at different times in each culture, and took over the population. While the specific genetic mechanism responsible for control of M protein expression was not a main focus of this work, it is a subject of great interest. Reduction in M protein expression has previously been observed following prolonged nasal carriage of streptococci, a phenomenon termed phase variation [61–64]. It is likely that similar mechanisms are responsible for the reduction in M protein expression observed here. The sortase mutant model system offers unique advantages for further exploration of M protein regulation, due to the strong selective pressure against M protein expression. Such experiments are currently under way.

Despite the study of sortase mutants in various other Gram-positive organisms, morphological defects of the type described here were generally not observed; sortase mutants typically display normal growth and morphology [8, 32, 35, 38]. A notable exception is *Actinomyces oris* where deletion of sortase is lethal due to accumulation of a surface glycoprotein in the membrane [65]. The phenotype of passaged *S*. *pyogenes* sortase mutants that lost M protein expression resembled that of sortase mutants in other organisms, in that no morphological aberrations or reduction in growth rate were seen despite the absence of surface protein anchoring [8, 32, 35, 38]. Interestingly, a reduction in surface protein expression was also observed in sortase mutants of *S*. *aureus* (although the mechanism was not described) [2], and *Listeria monocytogenes* [66]. In the latter case, the mRNA encoding surface proteins was unchanged, suggesting that a post-transcriptional mechanism may regulate the expression level or stability of surface proteins, and may adjust their expression level in response to changes in cellular processing capacity [66]. It is therefore not clear whether expression of a wild type level of surface proteins in these sortase mutants could also be deleterious. This is further supported by the observation that overexpression of certain surface proteins in *S*. *aureus* sortase mutant is toxic [2].

## Conclusions

Sortase is considered a promising target for the development of anti-infective therapy, since inhibitors of sortase could drastically reduce the pathogenicity of Gram-positive bacteria by preventing the anchoring of virulence factors to their wall [37, 67–70]. This is based on numerous observations showing that sortase mutants are much less pathogenic than a wild type bacterium [27–38, 71, 72]. Since most sortase mutants do not display notable growth defects in laboratory medium, sortase inhibitors are generally believed to affect pathogenicity but not viability [37]. Based on the results presented here however, it is likely that sortase inhibitors would not only prevent the anchoring of virulence factors to the wall of *S*. *pyogenes* but also lead to aberrant morphology, permeabilized membrane, increased sensitivity to AMPs, and an inability to grow in the blood environment. Although in laboratory medium selective pressure eventually led to variants with reduced surface protein expression and thus less morphological defects, the type of selective pressures found in the host environment are very different. The low fitness of sortase-inhibited cells in the host environment could lead to their elimination before they have time to adjust. Furthermore, variants with reduced surface protein expression would remain exposed to phagocytic killing, and would therefore not be as likely to take over the population. Small-molecule sortase inhibitors may therefore be more effective antibacterial agents than previously believed.

## Materials and Methods

### Ethics Statement

All the procedures involving human subjects were performed in accordance with a protocol approved by the Rockefeller University Institutional Review Board, IRB number VFI-0790; written consent was obtained from all participants.

### Bacterial strains, plasmids, and culture conditions

*S*. *pyogenes* strain D471 (serotype M6) was from the Rockefeller University collection. AR01 is a variant of D471, in which the *srtA* gene was replaced with an erythromycin resistance cassette [39]. Construction of the sortase expression plasmid, pAR107, is described elsewhere [39]. JRS75 is an M protein mutant of JRS4, a streptomycin resistant variant of D471 [73]. We obtained JRS75 immediately after its creation, and have the specific D471 strain used to make JRS4. A recent genomic sequencing comparison of D471 and JRS4 however, revealed 5 SNPs in addition to the one conferring streptomycin resistance [74]. While these may represent later accumulation of genetic variation, the possibility that these SNPs also exist in the JRS75 variant used in this study should be considered in the interpretation of results involving this strain. *S*. *pyogenes* strains were grown in Todd-Hewitt medium (Difco) supplemented with 1% yeast extract (Fisher Scientific), stationary at 37°C. Erythromycin was used at 15 μg/ml, and spectinomycin was used at 120 μg/ml where appropriate.

### Fractionation of *S*. *pyogenes* cells and Western blot analysis

Fractionation of the cells, and Western blot analysis were carried out essentially as previously described [39]. Briefly, overnight cultures were diluted 1:100 into fresh medium and grown to OD_600_ 0.5. The cells were harvested and the supernatant was precipitated with trichloroacetic acid (TCA) at a final concentration of 5% (“supernatant” fraction). The pellet was washed and resuspended in 200 μl PBS 30% raffinose containing 300 U/ml PlyC [75] for 15 min at room temperature. The resulting spheroplasts were harvested by centrifugation and the supernatant was precipitated with 5% TCA (“wall” fraction). The spheroplast pellet was washed with PBS 30% raffinose and resuspended in 60 μl Laemmli buffer (“spheroplast fraction”). The protein pellets resulting from TCA precipitation were washed with 1 ml acetone and resuspended in 60 μl Laemmli buffer. Samples were separated by 10% or 4–15% SDS PAGE, and transferred to polyvinylidene fluoride membranes. The M protein specific 10B6 monoclonal antibody [76] was used at a 1:5,000 dilution, and the anti-GAPDH monoclonal antibody was used at a 1:150 dilution. Goat anti-mouse horseradish peroxidase conjugate was used at a 1:5000 dilution. SfbI-specific rabbit serum [77], adsorbed on *S*. *pyogenes* SF370, which does not contain an *sfbI* gene [48], was used at a 1:2000 dilution. Biotinylated goat anti-rabbit antibody (Sigma) was used at a 1:5000 dilution, and streptavidin horseradish peroxidase conjugate (Sigma) was used at a 1:5000 dilution.

### Immunofluorescence Microscopy

Fluorescent microscopy procedures were carried out as previously described [17]. Briefly, *S*. *pyogenes* cells were fixed for 15 min at room temperature, and 30 min on ice, using 2.6% Paraformaldehyde, 0.012% glutaraldehyde, and 30 mM phosphate buffer pH 7.4. Fixed cells were washed with PBS, and attached to polylysine-coated cover glass. The cells were washed and blocked for 15 min with 10% normal goat serum. Primary antibodies and fluorescent conjugates were diluted in PBS containing 2% BSA and 1% gelatin. Each was incubated with the cells for 1 h at room temperature. Slides were mounted in 50% glycerol and 0.1% *p*-phenylenediamine in PBS pH 8. Phase-contrast and fluorescence microscopy were performed using a Nikon Eclipse E400 microscope, equipped with a Nikon 100×/1.25 oil immersion lens, and a Retiga EXi fast 1394 camera (QImaging). Images were captured and processed using QCapture Pro version 5.1.1.14 software (QImaging). Deconvolution images were obtained using a DeltaVision image restoration microscope (Applied Precision/Olympus) equipped with CoolSNAP QE cooled CCD camera (Photometrics). An Olympus 100×/1.40 NA, UPLS Apo oil immersion objective was used in conjunction with a 1.5× optovar. Z-stacks were taken at 0.15 μm intervals. Images were deconvolved using the SoftWoRx software (Applied Precision/DeltaVision), and corrected for chromatic aberrations. The M-protein-specific 10B6 monoclonal antibody [76] was used at a 1:1000 dilution. SfbI-specific rabbit serum and pre-immune serum [77], were used at a 1:1000 dilution. Goat anti-mouse IgG Rhodamine red conjugate (Jackson ImmunoResearch) was used at a 1:1000 dilution. Goat anti-rabbit IgG FITC conjugate (Sigma) was used at a 1:1000 dilution. Wheat germ agglutinin (WGA) Marina Blue conjugate (Invitrogen) was used at 5 μg/ml.

### Sortagging

Log phase cells of the strains D471, AR01, AR01+pAR107, and passaged AR01, were fixed and attached to microscope slides. The cells were gently permeabilized with PlyC to allow sortase to reach LPXTG motifs found beneath the surface of the cell wall as previously described [39]. The cells were then blocked with sortagging blocking buffer (100 mM tris pH 7.5, 150 mM NaCl, 2% BSA, 1% gelatin, 0.5 M glycine, 0.5 mM pentaglycine). Sortagging reactions were done in sortagging buffer (30 mM HEPES pH 7.4, 5 mM CaCl_2_, 1 mM dithiothreitol, 50 mM glycine) containing 40 μM pentaglycine-biotin (produced at the Rockefeller University Proteomics Resource Center), in the presence or absence of 240 μg/ml recombinant *S*. *aureus* SrtA (see S1 Materials and Methods); each slide was incubated in a moist chamber with 20 μl reaction mixture for 1 h at 37°C. Cells were thoroughly washed with PBS, and incubated with streptavidin FITC conjugate (1:1000 dilution), for 1 h at room temperature. Slides were mounted and DeltaVision images were obtained as described in the immunofluorescence microscopy section.

### SYTOX green assay

*S*. *pyogenes* were diluted 1:50 from an overnight culture to fresh TH+Y medium (containing spectinomycin for AR01+ pAR107), and grown to OD_600_ 0.5. SYTOX green (Life technologies) was added to a final concentration of 5 μM and the cultures were left at room temperature for 30 minutes. The cells were washed once with PBS and mounted in PBS. Phase-contrast and fluorescence microscopy were performed immediately as described above.

### Electron microscopy

For transmission electron microscopy, log phase cultures were harvested and resuspended in 0.1 M cacodylate buffer pH 7.4 containing 2.5% glutaraldehyde overnight at 4°C. Samples were then spun for 10 min at 3200 g at 4°C to create a compact pellet, and were post-fixed with 1% osmium tetroxide, stained en bloc with 0.5% uranyl acetate, dehydrated by a graded series of ethanol, infiltrated with EMBed812 resin, and embedded with the resin. After polymerization, ultrathin sections (70 nm thickness) were cut, stained with 2% uranyl acetate and 1% lead citrate, and examined under an electron microscope (100CX JEOL, Tokyo, Japan) with a digital imaging system (XR41-C, Advantage Microscopy Technology Corp, Danver, MA). For scanning electron microscopy studies, cells were attached to polylysine-coated cover slips and fixed in 2.5% glutaraldehyde at 4°C overnight. The cells were then treated with 1% osmium tetroxide in 0.1 M cacodylate buffer pH 7.4 for 1 h, dehydrated using graded ethanol solutions, and critical-point dried. The slides were coated with a thin gold-palladium layer using a Desk IV coater (Denton Vacuum). Images were obtained using a LEO 1550 scanning electron microscope, with field-emission electron gun.

### Capture ELISA

The wells of a 96-well microtiter plate were coated overnight at 4°C with 100 μl of 5 μg/ml 10B6 monoclonal anti-M protein antibody [76], or with 0.625 μg/ml fibronectin (Sigma) for the detection of SfbI. The plates were washed three times with distilled water and blocked with 100 μl sample buffer (PBS containing 1% BSA, 1% gelatin, and 0.02% sodium azide) at room temperature for 15 min, and then topped off with wash buffer (10 mM sodium phosphate, 150 mM NaCl, 0.05% Brij-35, 0.02% sodium azide) for additional 15 min. Samples were prepared by spinning 1 ml overnight culture, separating the supernatant, and lysing the pellet in 1 ml PBS containing 6 U/ml PlyC. Samples were initially diluted fivefold in sample buffer, and were then subjected to six threefold serial dilutions, which were all tested on the plate. M protein plates included a standard of purified recombinant M protein at the following concentrations: 500 ng/ml, 100 ng/ml, 20 ng/ml, 5 ng/ml, 2 ng/ml, 1 ng/ml, 0.5 ng/ml, 0.25 ng/ml, and 0.01 ng/ml. The standard for SfbI plates consisted of a D471 lysate from a single log phase culture, frozen in aliquots, and serially diluted as described above (allowing for normalization of the data, but not quantification of the protein). Plates containing lysates and standards were incubate at 37°C for 3 h, and washed. All wash steps consisted of three washes with distilled water, and two with wash buffer. The plates were then incubated at 4°C overnight with 100 μl/well rabbit polyclonal serum specific for M protein or SfbI, at 1:2000 and 1:4000 dilutions respectively. The plates were washed and incubated for 3 h at 37°C with 100 μl/well goat anti rabbit, alkaline phosphatase conjugate, diluted 1:4000 in sample buffer for M protein, or 1:2000 for SfbI. The plates were then washed and developed for 15 min at 37°C with 200 μl/well of 10% diethanolamine, 0.5 mM MgCl_2_, pH 10, containing 1 μg/ml p-nitrophenyl phosphate. Absorption at 405 nm was measured using a SpectraMax Plus plate reader (Molecular Devices).

### LL-37 killing assay

Overnight cultures of the various streptococcal strains were diluted 1:50 into 5 ml TH+Y (containing spectinomycin for AR01+pAR107), and grown to OD_600_ 0.5. The cultures were diluted 1:10^5^ and 100 μl of the diluted sample was mixed with 100 μl medium containing twice the final concentration of peptide. For overnight endpoint assays, the samples were placed in a 96-well plate and incubated stationary for 16 h at 37°C, at which point samples were resuspended using a multichannel pipette, and absorption at 600 nm was measured using a SpectraMax Plus plate reader (Molecular Devices); experiments were carried out in triplicates. For the CFU-based assays, samples were prepared in a similar manner, however they were rotated in microfuge tubes for 3 h at 37°C, and then serially diluted and plated for CFU quantification; experiments were done in duplicates. The CFU count of each peptide-treated sample was divided by that of the equivalent untreated sample to yield the final result. Data analysis was done using Prism version 5.0c (GraphPad Software).

### Direct bactericidal assay

Bactericidal assays were performed with modification from Lancefield *et al*. [56]. Blood was collected in heparinized tubes from healthy volunteers that tested negative for *S*. *pyogenes* M6-serotype antibodies. Overnight cultures of the various streptococcal strains were diluted 1:50 into 5 ml TH+Y, and grown to OD_600_ 0.2. Reaction mixtures were prepared in 2 ml screw-cap tubes (SARSTEDT). To each reaction tube were added 100 μl TH+Y, and 100 μl streptococcal culture, diluted 1:10^5^ (unless otherwise noted) in TH+Y. Then were added 400 μl heparinized blood (two sets of tubes), or 400 μl blood that was incubated for 20 min on ice with 30 μM cytochalasin B (bringing the final concentration in the reaction tube to 20 μM), or 400 μl plasma that was freshly prepared from the same blood sample by centrifugation, or 400 μl TH+Y. For AR01+pAR107 spectinomycin was included at a final concentration of 100 μg/ml. The tubes were rotated end-over-end for 3 h at 37°C, except for one of the sets of unmodified blood tubes, which was left stationary at the same conditions. Following incubation, CFU counts were determined by serially diluting the samples 10-fold, and plating on TH+Y plates. Experiments were done in duplicates, with two technical duplicates for each of the biological ones; the experiment was repeated three times. Technical duplicates were averaged, and the results were analyzed using Prism version 5.0c (GraphPad Software).

## Supporting Information

S1 Fig The sortase mutant AR01 grows slower than wild type D471; passaged AR01 grows similarly to D471. **A** D471, AR01, and five different AR01 variants that were passaged in laboratory medium for 6 days were diluted 1:100 from an overnight stock into fresh TH+Y medium. OD_600_ values were measured every 30 minutes. **B** The OD_600_ values from the log-phase growth stage of D471 and AR01 were overlaid, and are presented in linear and log scales.
(TIFF)

S2 Fig Alignment of CWS from *S*. *pyogenes* MGAS10394 surface proteins. The genome of *S*. *pyogenes* MGAS10394 was scanned for the presence of proteins containing a conserved CWS, composed of an LPXTG motif, a hydrophobic stretch, and a few C-terminal positively charged residues. The CWS were aligned using MegAlign (DNASTAR). Known SrtB substrates are labeled with an asterisk.
(TIF)

S3 Fig Signal sequences of *S*. *pyogenes* MGAS10394 surface proteins. The genome of *S*. *pyogenes* MGAS10394 was scanned for the presence of proteins containing a conserved CWS, composed of an LPXTG motif, a hydrophobic stretch, and a few C-terminal positively charged residues (alignment of these CWS is presented in S1 Fig). The location of the signal sequence and the predicted signal peptidase cleavage site were analyzed using SignalP 4.1 (based on annotated protein sequences). Signal peptides were divided into two groups based on the presence of a YSIRK G/S motif (including a partial motif), and the signal sequences within each group were aligned. A signal sequence was not detected within the annotated protein sequence of SclB (M6_Spy0797) and Cpa (M6_Spy0159), and they were excluded form this analysis (see text for details).
(TIF)

S4 Fig Passage of the sortase mutant AR01 in TH+Y results in a return to normal chain organization and the loss of M protein expression. Wild type *S*. *pyogenes* D471, sortase mutant AR01, complemented AR01+pAR107, and 10 separate variants of AR01 that were passaged 6 times in TH+Y, were diluted from overnight cultures 1:100 into fresh media (containing spectinomycin for AR01+pAR107). Log phase cells were fixed, and processed for fluorescence microscopy as described in the Materials and Methods section. Specific antibodies were used to label M protein (red) and SfbI (green). The cell wall was stained with WGA marina blue (blue). Immunofluorescence and phase-contrast microscopy images are presented.
(TIF)

S5 Fig The *S*. *pyogenes* sortase mutant is under selective pressure leading to the loss of M protein expression—dot blot analysis of AR01. Ten colonies of the sortase mutant AR01 (A-J) were passaged for 6 consecutive days in TH+Y. A sample was collected from the cultures at each passage, and separated into supernatant and pellet. The pellet was lysed with PlyC, and both fractions were serially diluted and analyzed by semi-quantitative dot-blot, using the monoclonal antibodies 10B6 (specific to M protein) and 1A3 (specific for GAPDH) as loading control.
(TIF)

S6 Fig Control for SfbI-specific ELISA—the assay does not recognize the *sfbI*-negative M1 strain SF370. *S*. *pyogenes* strains AR01 and SF370 (a serotype M1 strain lacking a *sfbI* gene) were grown overnight at 37°C, and processed for capture ELISA in a manner similar to that described in Fig 5B. Raw absorbance data is presented for all the samples in a serial 3-fold dilution set. Duplicate assays are presented with mean and SEM values.
(TIFF)

S7 Fig Independently isolated *S*. *pyogenes* sortase mutants are all under selective pressure leading to the loss of M protein. Two colonies of each of the sortase mutant strains AR01, AR01.1, AR01.2, AR01.3, and AR01.4, were passaged for 6 consecutive days in TH+Y. A sample was collected from the cultures at each passage, and separated into supernatant and pellet. The pellet was lysed with PlyC, and both fractions were serially diluted and analyzed by semi-quantitative dot-blot, using the monoclonal antibodies 10B6 (specific to M protein) and 1A3 (specific for GAPDH) as loading control.
(TIF)

S8 Fig The sortase mutant, but not the passaged strain, displays increased membrane permeability—additional images. This figure provides additional data to that presented in Fig 7. Wild type D471, sortase mutant AR01 (original stock), complemented AR01+pAR107, and a low-M passaged variant of AR01, were diluted 1:50 from an overnight culture and grown to OD_600_ 0.5. The cells were incubated with 5 mM SYTOX green for 30 min at room temperature, washed with PBS, and immediately imaged. This experiment was repeated on three separate days, and a large field is presented from each experiment. The images are presented in their original resolution, allowing zooming in on individual cells. The scale bar represents 5 μm.
(TIF)

S9 Fig M protein and sortase double mutant is not highly susceptible to killing in plasma. The M-protein-negative strain JRS75 and its sortase-negative derivate AR03.1 were grown to OD_600_ 0.2, diluted 1:10^5^, and incubated in the following conditions: 1) rotated in blood, 2) stationary in blood (phagocytes sink to the bottom), 3) rotated in blood + 20 μM cytochalasin B (inhibits phagocytosis), 4) rotated in plasma, 5) rotated in TH+Y. Blood and plasma were from a healthy donor who tested negative for antibodies specific to M protein serotype 6. Following 3 h incubation at 37°C, samples were serially diluted 10-fold, and plated on TH+Y for CFU quantification; results are presented as CFU_final_/CFU_start_. Experiments were done in triplicates and error bars represent SEM. P values were calculated using t-test; “ns” denotes no statistical significance. # symbol denotes that one or more data points in the group were below detection level.
(TIFF)

S1 Materials and Methods (DOCX)

S1 Table Primer used in this work. (DOCX)

## Abbreviations

AMP: antimicrobial peptide
BSA: bovine serum albumin
CFU: colony forming unit
CWS: cell wall sorting signal
PBS: phosphate buffered saline
TCA: trichloroacetic acid
WGA: wheat germ agglutinin
