# Supplementary figures and images for: Streptococcus pyogenes Sortase Mutants Are Highly Susceptible to Killing by Host Factors Due to Aberrant Envelope Physiology
Source: PLoS One. 2015 Oct 20;10(10):e0140784. doi: 10.1371/journal.pone.0140784 (PMC4617865; doi:10.1371/journal.pone.0140784)

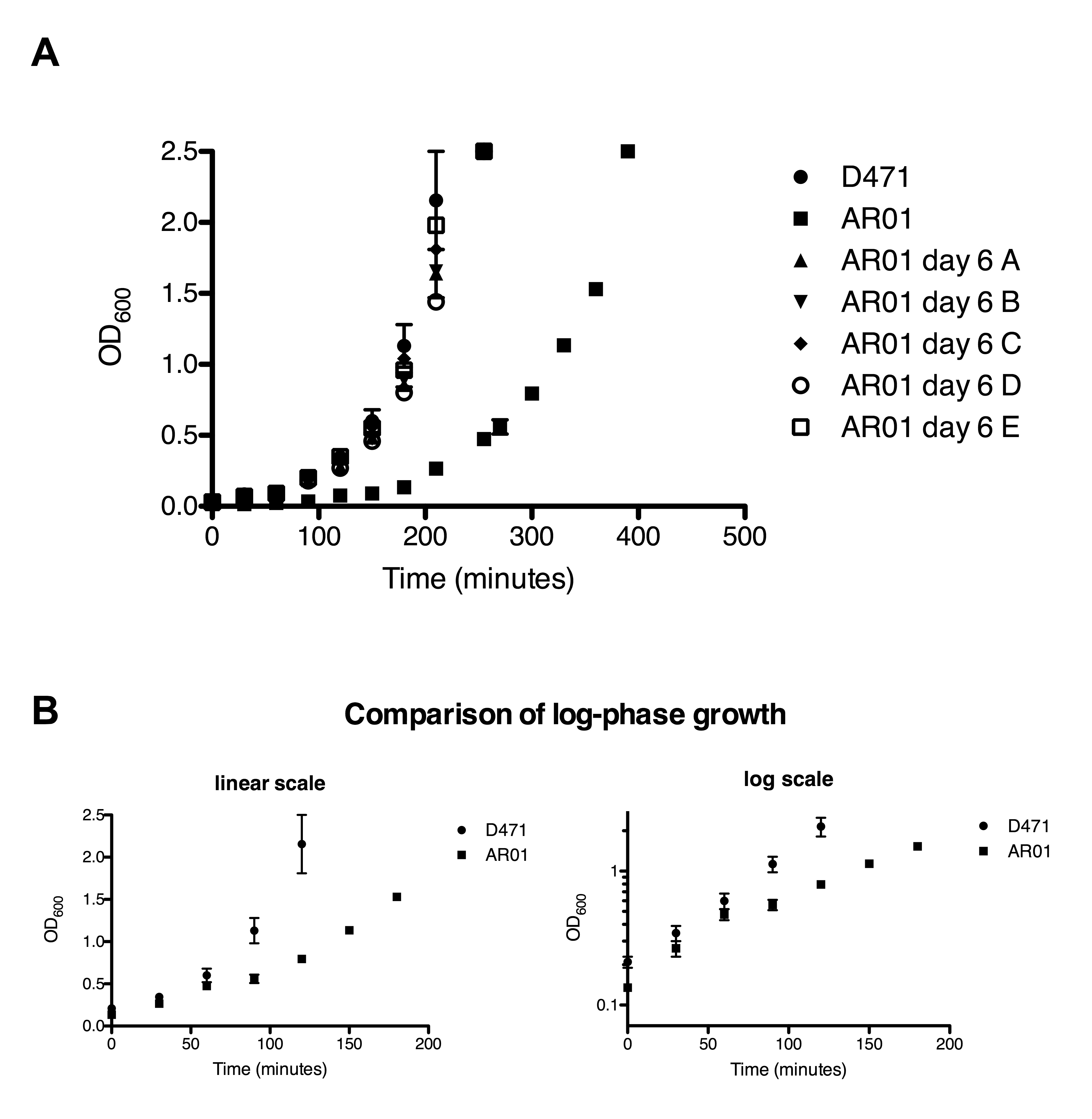

Supplement: S1 Fig — A D471, AR01, and five different AR01 variants that were passaged in laboratory medium for 6 days were diluted 1:100 from an overnight stock into fresh TH+Y medium. OD600 values were measured every 30 minutes. B The OD600 values from the log-phase growth stage of D471 and AR01 were overlaid, and are presented in linear and log scales. (TIFF) [file pone.0140784.s001.tiff]

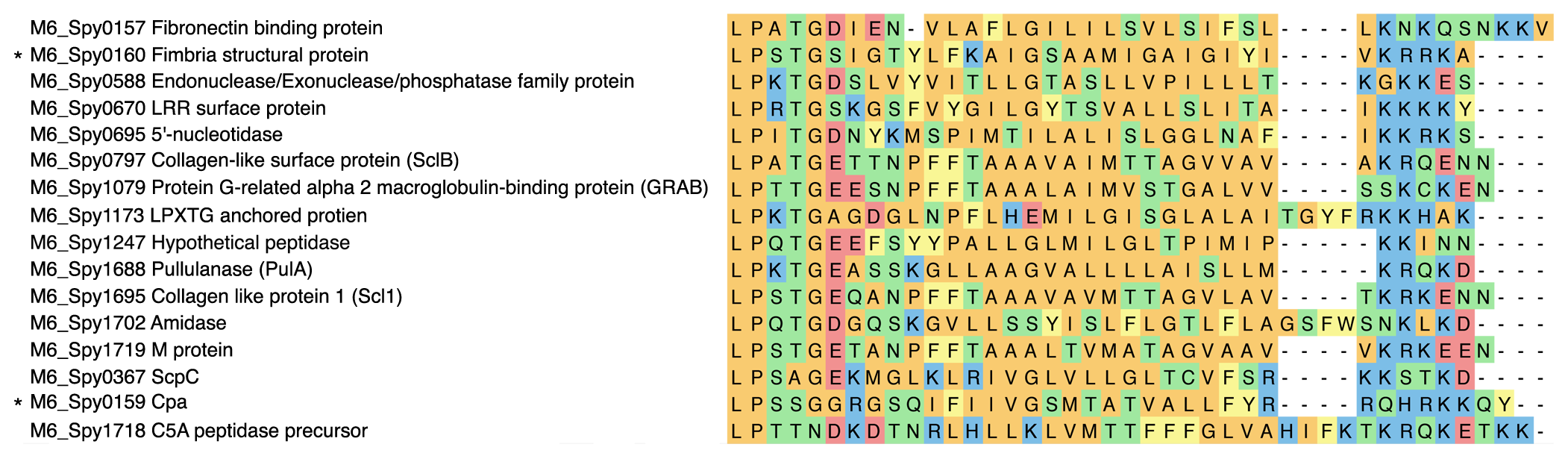

Supplement: S2 Fig — The genome of S. pyogenes MGAS10394 was scanned for the presence of proteins containing a conserved CWS, composed of an LPXTG motif, a hydrophobic stretch, and a few C-terminal positively charged residues. The CWS were aligned using MegAlign (DNASTAR). Known SrtB substrates are labeled with an asterisk. (TIF) [file pone.0140784.s002.tif]

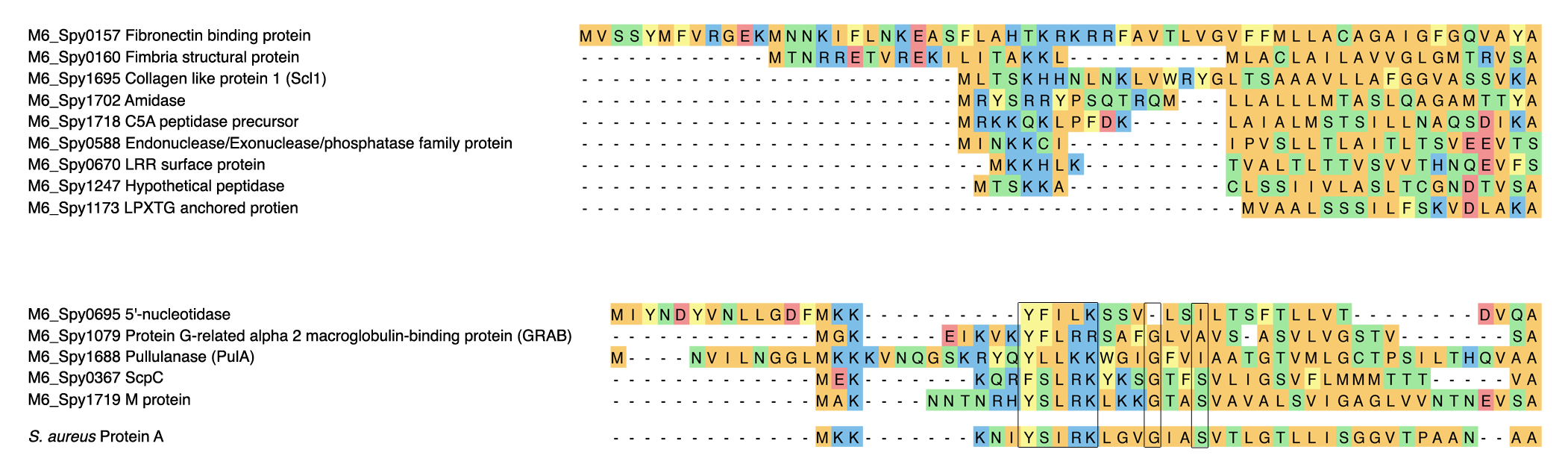

Supplement: S3 Fig — The genome of S. pyogenes MGAS10394 was scanned for the presence of proteins containing a conserved CWS, composed of an LPXTG motif, a hydrophobic stretch, and a few C-terminal positively charged residues (alignment of these CWS is presented in S1 Fig). The location of the signal sequence and the predicted signal peptidase cleavage site were analyzed using SignalP 4.1 (based on annotated protein sequences). Signal peptides were divided into two groups based on the presence of a YSIRK G/S motif (including a partial motif), and the signal sequences within each group were aligned. A signal sequence was not detected within the annotated protein sequence of SclB (M6_Spy0797) and Cpa (M6_Spy0159), and they were excluded form this analysis (see text for details). (TIF) [file pone.0140784.s003.tif]

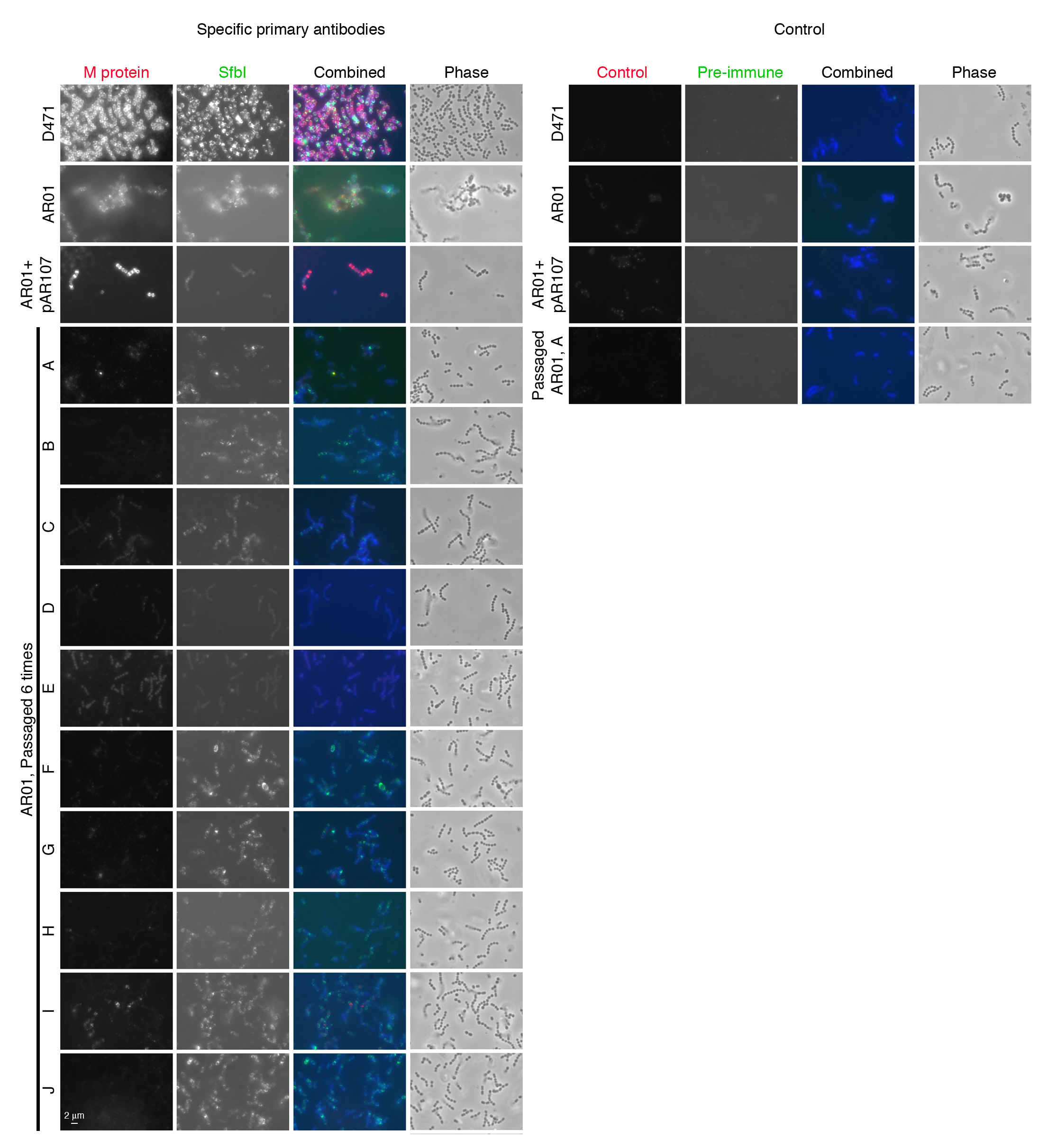

Supplement: S4 Fig — Wild type S. pyogenes D471, sortase mutant AR01, complemented AR01+pAR107, and 10 separate variants of AR01 that were passaged 6 times in TH+Y, were diluted from overnight cultures 1:100 into fresh media (containing spectinomycin for AR01+pAR107). Log phase cells were fixed, and processed for fluorescence microscopy as described in the Materials and Methods section. Specific antibodies were used to label M protein (red) and SfbI (green). The cell wall was stained with WGA marina blue (blue). Immunofluorescence and phase-contrast microscopy images are presented. (TIF) [file pone.0140784.s004.tif]

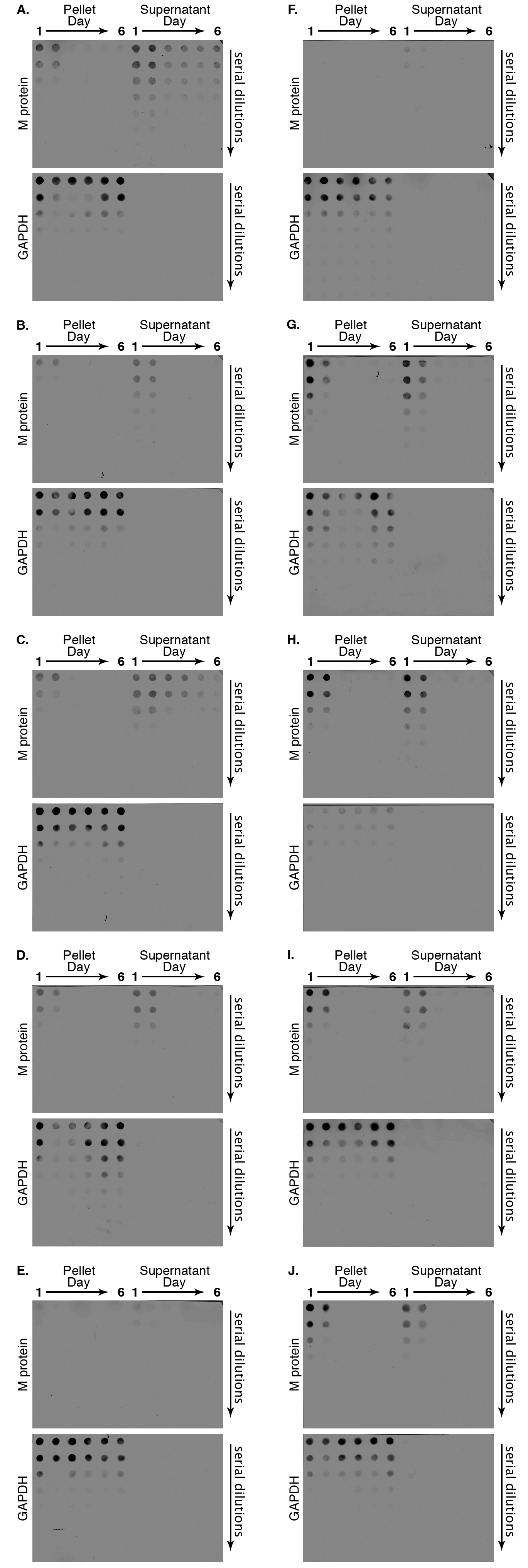

Supplement: S5 Fig — Ten colonies of the sortase mutant AR01 (A-J) were passaged for 6 consecutive days in TH+Y. A sample was collected from the cultures at each passage, and separated into supernatant and pellet. The pellet was lysed with PlyC, and both fractions were serially diluted and analyzed by semi-quantitative dot-blot, using the monoclonal antibodies 10B6 (specific to M protein) and 1A3 (specific for GAPDH) as loading control. (TIF) [file pone.0140784.s005.tif]

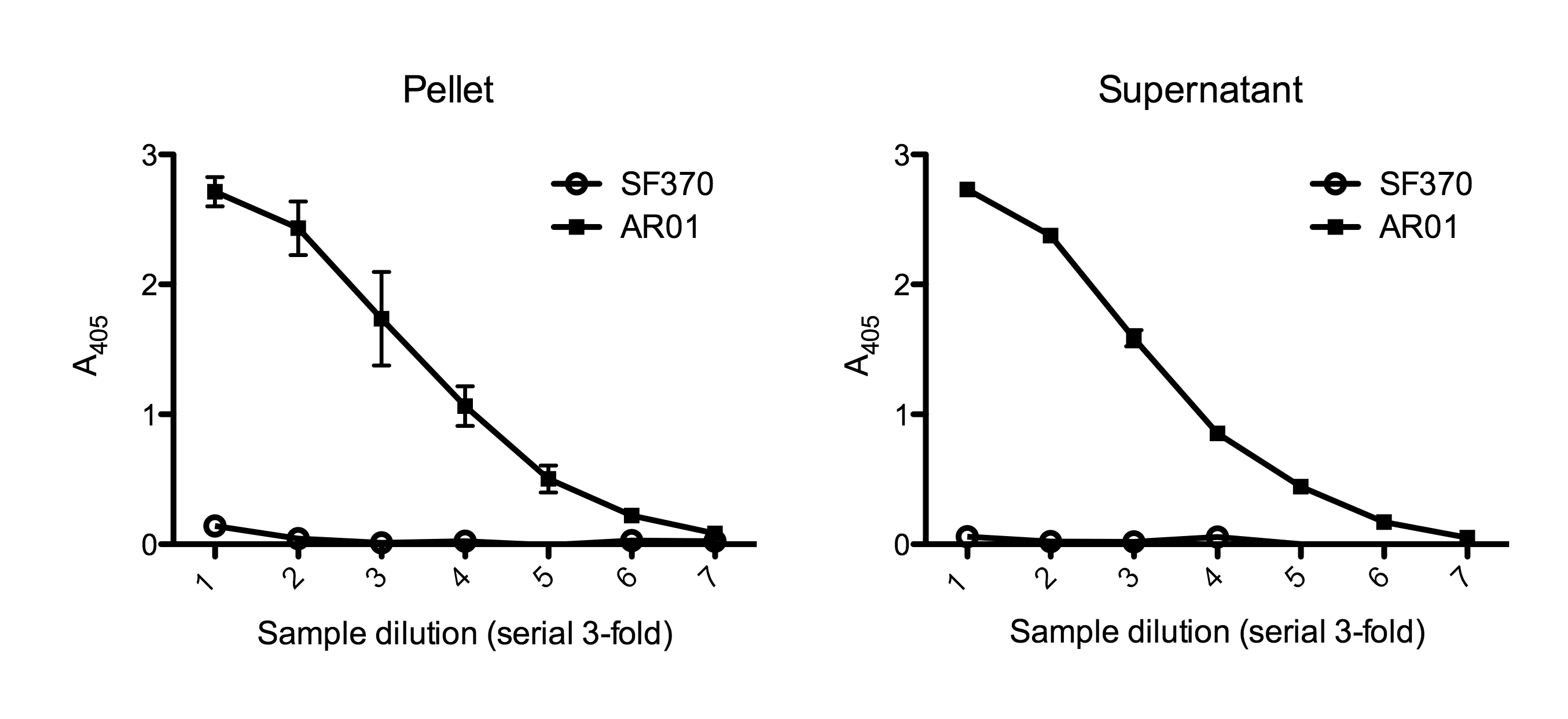

Supplement: S6 Fig — S. pyogenes strains AR01 and SF370 (a serotype M1 strain lacking a sfbI gene) were grown overnight at 37°C, and processed for capture ELISA in a manner similar to that described in Fig 5B. Raw absorbance data is presented for all the samples in a serial 3-fold dilution set. Duplicate assays are presented with mean and SEM values. (TIFF) [file pone.0140784.s006.tiff]

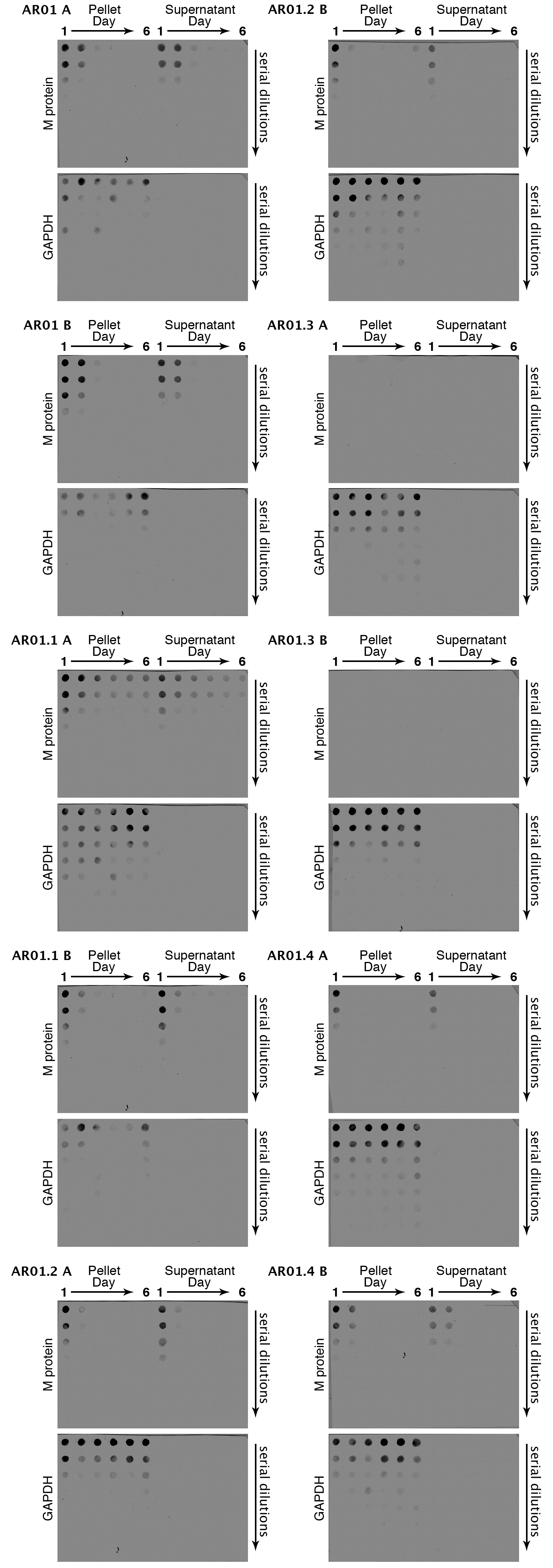

Supplement: S7 Fig — Two colonies of each of the sortase mutant strains AR01, AR01.1, AR01.2, AR01.3, and AR01.4, were passaged for 6 consecutive days in TH+Y. A sample was collected from the cultures at each passage, and separated into supernatant and pellet. The pellet was lysed with PlyC, and both fractions were serially diluted and analyzed by semi-quantitative dot-blot, using the monoclonal antibodies 10B6 (specific to M protein) and 1A3 (specific for GAPDH) as loading control. (TIF) [file pone.0140784.s007.tif]

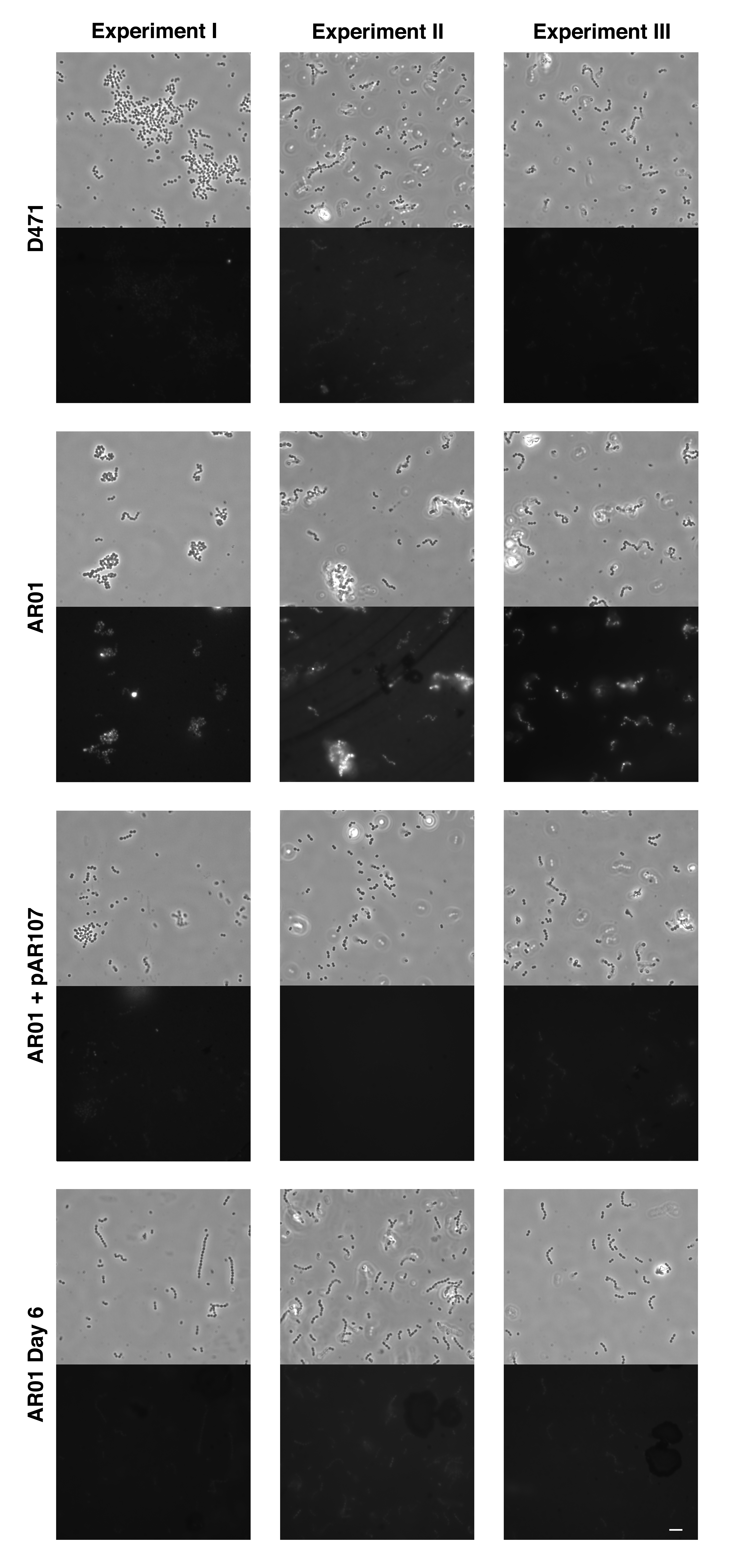

Supplement: S8 Fig — This figure provides additional data to that presented in Fig 7. Wild type D471, sortase mutant AR01 (original stock), complemented AR01+pAR107, and a low-M passaged variant of AR01, were diluted 1:50 from an overnight culture and grown to OD600 0.5. The cells were incubated with 5 mM SYTOX green for 30 min at room temperature, washed with PBS, and immediately imaged. This experiment was repeated on three separate days, and a large field is presented from each experiment. The images are presented in their original resolution, allowing zooming in on individual cells. The scale bar represents 5 μm. (TIF) [file pone.0140784.s008.tif]

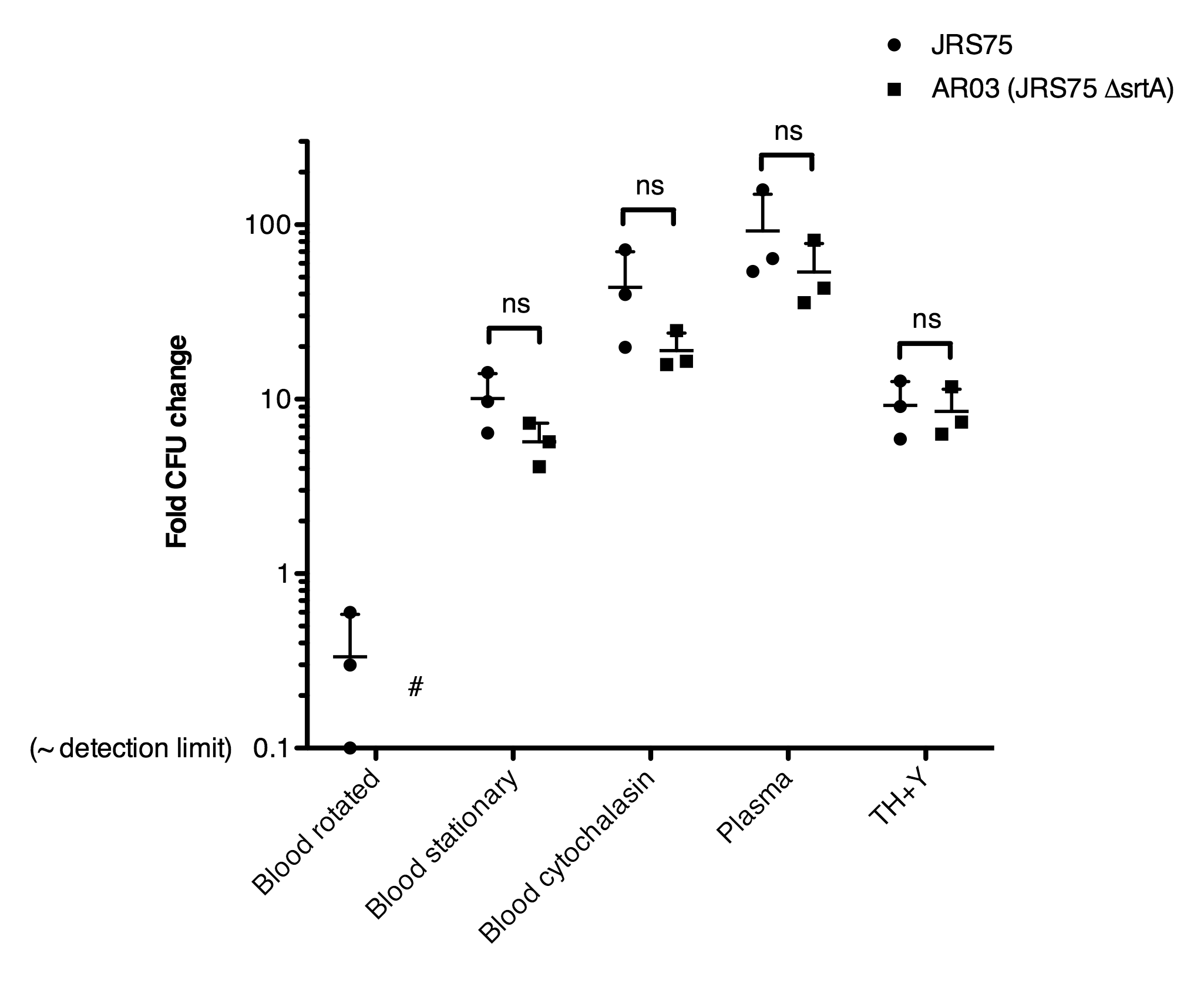

Supplement: S9 Fig — The M-protein-negative strain JRS75 and its sortase-negative derivate AR03.1 were grown to OD600 0.2, diluted 1:105, and incubated in the following conditions: 1) rotated in blood, 2) stationary in blood (phagocytes sink to the bottom), 3) rotated in blood + 20 μM cytochalasin B (inhibits phagocytosis), 4) rotated in plasma, 5) rotated in TH+Y. Blood and plasma were from a healthy donor who tested negative for antibodies specific to M protein serotype 6. Following 3 h incubation at 37°C, samples were serially diluted 10-fold, and plated on TH+Y for CFU quantification; results are presented as CFUfinal/CFUstart. Experiments were done in triplicates and error bars represent SEM. P values were calculated using t-test; “ns” denotes no statistical significance. # symbol denotes that one or more data points in the group were below detection level. (TIFF) [file pone.0140784.s009.tiff]
