# Supplementary material for: Streptococcus pyogenes Sortase Mutants Are Highly Susceptible to Killing by Host Factors Due to Aberrant Envelope Physiology
Source: PLoS One. 2015 Oct 20;10(10):e0140784. doi: 10.1371/journal.pone.0140784 (PMC4617865; doi:10.1371/journal.pone.0140784)
Supplement: S1 Materials and Methods — (DOCX) [file pone.0140784.s010.docx]

**Supplementary Materials and Methods**

**Dot Blot analysis.**  For semi-quantitative analysis, samples were prepared as described for the ELISA assay, but using two-fold serial dilution. For detection of M protein expression in colonies isolated following blood rotation, a cell pellet derived from 50 μl overnight culture was suspended in 100 μl PBS, lysed with PlyC, and loaded on a single dot. Samples were applied to a nitrocellulose membrane using a dot-blot vacuum apparatus. The blots were dried briefly and blocked with PBS containing 5% dried milk and 0.1% Tween 20 (PBS-DT) for 2 hours at room temperature with shaking, and washed with tris buffered saline (137mM NaCl, 20mM tris pH 7.5) containing 0.1% tween (TBS-T). Antibodies were diluted in PBS-DT and incubated at 4^o^C overnight with shaking. Blots were developed with nitro blue tetrazolium chloride (NBT) / 5-Bromo-4-chloro-3-indolyl phosphate (BCIP), or SuperSignal West Pico Chemiluminescent Substrate (Thermo).

**Cloning and purification of *Staphylococcus aureus* SrtA**

The DNA sequence encoding the *S. aureus* *srtA* gene, lacking its N-terminal transmembrane domain (amino acids 1-25), was amplified from genomic DNA of strain MW2 (community acquired MRSA), using primers 5_SA_srtA_SalI and 3_SA_srtA_NotI. The resulting PCR product was inserted into the SalI and NotI restriction sites of a modified pET21a plasmid (a gift from Dr. Erec Stebbins) yielding pAR203. This plasmid contains an open reading frame, in which a hexahistidine tag followed by a 3C protease cleavage site, are fused to the N-terminus of the soluble portion of SrtA.

An overnight culture of *Escherichia coli* BL21/pAR203 was diluted 1:100 into 800 ml of LB medium containing 100 μg/ml ampicillin and grown at 37°C with shaking. Upon reaching OD_600_ 0.6, the expression of H6-SrtA was induced with 1 mM IPTG for 3 hours. The cells were harvested, resuspended in 50 ml MCAC buffer (30 mM Tris pH 7.4, 0.5 M NaCl, 10% glycerol, 1 mM DTT), and homogenized. Cell debris was removed by centrifugation, and the supernatant was filtered through a 0.22 μm filter (Millipore). The cleared lysate was loaded on a NiNTA column equilibrated with MCAC buffer, and the column was washed thoroughly with MCAC containing 5 mM imidazole. SrtA was eluted with MCAC containing 100 mM imidazole, and positive fractions were concentrated using an Amicon ultrafiltration device fitted with a 10 kDa molecular weight cutoff membrane to a final concentration of 12 mg/ml.

**M protein expression vectors**

A DNA fragment encoding a new multi-cloning site region (MCS) EcoRI-HinDIII-BglII-SacII-PvuII-NcoI-SphI was created by heating and cooling the complementary primers 5_new_plzMCS and 3_new_plzMCS. This fragment was inserted between the *EcoRI* and *SphI* sites of pLZ12-spec [[1](#_ENREF_1)] yielding pAR161.

A DNA sequence encompassing the 5’-untranslated region of M protein and the 5’ region of the gene was amplified using primers 5_Mp_UTR_EcoRI and 3_Mp_Npart_BglII on template plasmid pJRS42.13 [[2](#_ENREF_2)], and inserted into the *EcoRI* and *BglII* sites of pAR161, yielding pAR163.

A region encoding the C-terminus of M protein was amplified using primers 5_Mp_Cpart_SacII and 3_Mp_Cpart_SphI on template pJRS42.13, and inserted into the *SacII* and *SphI* sites of pAR163, yielding pAR164.

Finally, three PCR products, encoding the central part of M protein up to, and including the LPXTG motif (each one encoding a different motif) were created using pJRS42.13 as a template; the sequence encoding the LPXTG motif was encoded on the 3’ primer. A PCR product using primer pair 5_M_mid_HindIII 3_M_LPSTGE_SacII was inserted into the *HinDIII* (found in the M protein coding sequence) and *SacII* sites of pAR164, yielding pAR186 (LPSTGE). A PCR product using primer pair 5_M_mid_HindIII 3_M_EGTSPL_SacII, was inserted into the *HinDIII* and *SacII* sites of pAR164, yielding pAR184 (EGTSPL). A PCR product using primer pair 5_M_mid_HindIII 3_M_TEPGSL_SacII, was inserted into the *HinDIII* and *SacII* sites of pAR164, yielding pAR185 (TEPGSL). Restriction sites were chosen in such a manner that the resulting amino acid sequence of M protein was not altered except for desired alterations in the LPXTG motif.

**Identification of proteins containing a CWS and YSIRK G/S motifs.** We used Python to scan the amino acid sequences of all annotated proteins in the MGAS10394 genome [[3](#_ENREF_3)] for the presence a conserved LPXTG motif at a distance of 25-45 amino acids from the C-terminus, allowing for up to one amino acid substitution within this motif. Resulting proteins were analyzed using the transmembrane prediction software DAS [[4](#_ENREF_4)] for the presence of a hydrophobic region following the LPXTG motif. The presence of positively charged residues at the C-terminus of the proteins was determined manually. This screen yielded 16 proteins that met all criteria for a CWS, of which 3 had an LPXTG motif with an amino acid substitution: Cpa (M6_Spy0159, LPSSG), ScpC (M6_Spy0367, LPSAG), and C5a peptidase (M6_Spy1718, LPTTN). Alignment of the CWS was done using DNASTAR MegAlign pro (S2 Fig). The 16 identified CWS were used as query sequences in a hidden Markov analysis [[5](#_ENREF_5)], in an attempt to find additional surface proteins that our initial screen might have missed, however no additional proteins containing a CWS were identified.

We identified a signal sequence in 14 of the 16 proteins, as annotated in the genome (S3 Fig), using SignalP 4.1 [[6](#_ENREF_6)], and established principles [[7](#_ENREF_7)]. Additionally, a possible in-frame signal sequence was identified upstream of the annotated start codon of *sclB* (M6_Spy0797), however we were not able to identify a signal sequence in *cpa* (M6_Spy0159) or in its upstream region. Nevertheless, *cpa* genes in other *S. pyogenes* genomes, such as NZ131 (M49) and MGAS315 (M3), have a possible 45 amino acid long signal sequence, although it is below the normal SignalP threshold. Sequencing the N-terminus and 5’-upstream regions of the two genes did not show any deviations from the published sequence (see below). Signal sequences were divided into two groups according to the presence of a YSIRK G/S motif (including partial motif) in their signal sequence. The two groups were aligned using DNASTAR MegAlign Pro (Fig. S3).

**Sequencing of genomic regions.** All PCR reactions were performed using Phusion High-Fidelity DNA Polymerase (New England BioLabs). Primers were from Fisher Scientific. PCR products were purified using QIAquick kit (QIAGEN). Sequencing reactions were carried out by GENEWIZ. Sequence analysis was performed using SeqMan pro (DNASTAR).

PCR products encompassing the N-terminus and upstream untranslated regions of SclB (M6_Spy0797) and Cpa (M6_Spy0159) were amplified from the MGAS10394 genome using primer pairs 5_SclB_up ­– 3_SclB_up, and 5_Cpa_up – 3_Cpa_up, respectively. Sequencing reactions were done using the same primer pairs.

1. Husmann LK, Scott JR, Lindahl G, Stenberg L. Expression of the Arp protein, a member of the M protein family, is not sufficient to inhibit phagocytosis of *Streptococcus pyogenes*. Infect Immun. 1995;63(1):345-8. Epub 1995/01/01. PubMed PMID: 7806375; PubMed Central PMCID: PMC172998.

2. Fischetti VA, Jones KF, Manjula BN, Scott JR. Streptococcal M6 protein expressed in *Escherichia coli*. Localization, purification, and comparison with streptococcal-derived M protein. J Exp Med. 1984;159(4):1083-95. Epub 1984/04/01. PubMed PMID: 6368734; PubMed Central PMCID: PMC2187285.

3. Banks DJ, Porcella SF, Barbian KD, Beres SB, Philips LE, Voyich JM, et al. Progress toward characterization of the group A Streptococcus metagenome: complete genome sequence of a macrolide-resistant serotype M6 strain. J Infect Dis. 2004;190(4):727-38. Epub 2004/07/24. doi: 10.1086/422697. PubMed PMID: 15272401.

4. Cserzo M, Wallin E, Simon I, von Heijne G, Elofsson A. Prediction of transmembrane alpha-helices in prokaryotic membrane proteins: the dense alignment surface method. Protein Eng. 1997;10(6):673-6. Epub 1997/06/01. PubMed PMID: 9278280.

5. Finn RD, Clements J, Eddy SR. HMMER web server: interactive sequence similarity searching. Nucleic Acids Res. 2011;39(Web Server issue):W29-37. Epub 2011/05/20. doi: 10.1093/nar/gkr367. PubMed PMID: 21593126; PubMed Central PMCID: PMC3125773.

6. Emanuelsson O, Brunak S, von Heijne G, Nielsen H. Locating proteins in the cell using TargetP, SignalP and related tools. Nat Protoc. 2007;2(4):953-71. Epub 2007/04/21. doi: 10.1038/nprot.2007.131. PubMed PMID: 17446895.

7. van Roosmalen ML, Geukens N, Jongbloed JD, Tjalsma H, Dubois JY, Bron S, et al. Type I signal peptidases of Gram-positive bacteria. Biochim Biophys Acta. 2004;1694(1-3):279-97. Epub 2004/11/18. doi: 10.1016/j.bbamcr.2004.05.006. PubMed PMID: 15546672.
