# Supplementary material for: Streptococcus pyogenes Sortase Mutants Are Highly Susceptible to Killing by Host Factors Due to Aberrant Envelope Physiology
Source: PLoS One. 2015 Oct 20;10(10):e0140784. doi: 10.1371/journal.pone.0140784 (PMC4617865; doi:10.1371/journal.pone.0140784)
Supplement: S1 Table — (DOCX) [file pone.0140784.s011.docx]

**S1 Table. Primer used in this work**

| Name | Sequence |
| --- | --- |
| 5_SA_srtA_SalI | CCCGTCGACAAACCACATATCGATAATTATCTTCACG |
| 3_SA_srtA_NotI | GGGGCGGCCGCTTATTTGACTTCTGTAGCTACAAAGATTTTAC |
| 5_new_plzMCS | AATTCCCCAAGCTTCCCAGATCTAAACCGCGGAAACAGCTGAAACCATGGAAAGCATG |
| 3_new_plzMCS | CTTTCCATGGTTTCAGCTGTTTCCGCGGTTTAGATCTGGGAAGCTTGGGG |
| 5_Mp_UTR_EcoRI | CGCGAATTCACAGCCTAGCCGCAGAAACTC |
| 3_Mp_Npart_BglII | CGCAGATCTAGTTTCCTTCATTGGTGCTTTGTTTTG |
| 5_Mp_Cpart_SacII | TCACCGCGGCAGCCCTTACTGTTATGG |
| 3_Mp_Cpart_SphI | CGCGCATGCTTAGTTTTCTTCTTTGCGTTTTACAAC |
| 5_M_mid_HindIII | AAAAAGCTTTAGAAGAAGCAAACAGCAAATTAG |
| 3_M_LPSTGE_SacII | CTGCCGCGGTGAAGAATGGGTTAG |
| 3_M_EGTSPL_SacII | CTGCCGCGGTGAAGAATGGGTTAGCTGTTAATGGTGATGTACCTTCCTGTCTCTTAGTTTCCTTCATTGGTGC |
| 3_M_TEPGSL_SacII | CTGCCGCGGTGAAGAATGGGTTAGCTGTTAATGAACCTGGTTCTGTCTGTCTCTTAGTTTCCTTCATTGGTGC |
| 5_SclB_up | TTATGAACGCGGGAAAATTATTG |
| 3_SclB_up | CGTCTTTACCATCCAGTAGTCTATCTTG |
| 5_Cpa_up | CGAAGTATACCTACTTCACTTGTGTCTG |
| 3_Cpa_up | TAAAAGTTAAGGTTGTTGTGATATCGTTTC |

- Restriction sites are marked with an underline.
